# Associations between on-farm factors and bulk tank SCC on Irish dairy farms

**DOI:** 10.1186/s13620-025-00300-8

**Published:** 2025-07-07

**Authors:** Alice Uí Chearbhaill, Pablo Silva Boloña, Eoin G. Ryan, Catherine I. McAloon, Conor G. McAloon, John Upton

**Affiliations:** 1 Teagasc, Animal & Grassland Research and Innovation Centre, Moorepark, Fermoy, Co., Cork, P61 C997 Ireland; 2 School of Veterinary Medicine, University College Dublin, Belfield, Dublin 4, Dublin, D04 W6F6 Ireland

**Keywords:** Parlour technologies, Milking management, Farm management, SCC control

## Abstract

**Background:**

This study describes associations between bulk tank somatic cell count (BTSCC) and farm management practices, parlour management practices and implemented technologies, milking management practices, somatic cell count (SCC) control strategies, and farmer demographics and attitudes around SCC management using a sample of Irish dairy farms.

**Results:**

This paper utilised a pre-existing dataset from a farm management and technology survey of 376 commercial Irish dairy farms conducted in 2022. Five mixed models were used to examine associations between variables in each of the five survey sections and log-10 transformed BTSCC (log10BTSCC). Seasonal calving patterns, family members milking alongside survey respondents, and keeping of mastitis treatment records were associated with lower log10BTSCC. Parlour technologies such as automatic cluster removers and automatic washers on the milking machine were associated with significantly reduced log10BTSCC, whereas the presence of backing gates and straight breast rails were associated with increased log10BTSCC. Fore-milking, pre-milking udder preparation and post-milking teat disinfection contributed to lower log10BTSCC. Advice sought from veterinary professionals regarding SCC, multi-faceted approaches to selective dry cow therapy decisions, and utilisation of results from multiple milk recordings were also associated with significantly decreased log10BTSCC.

**Conclusions:**

In this study, we successfully established associations between log10BTSCC and farm management practices, parlour management practices and implemented technologies, milking management practices, SCC control strategies, and farmer demographics and attitudes around SCC management. We identified scope for further research on many of the aspects found to be associated with log10BTSCC in this study, particularly in the areas of cow positioning within parlours, fore-milking practices, milk recording, and means of disseminating SCC advice to farmers, particularly around the topics of parlour hygiene and selective dry cow therapy.

**Supplementary Information:**

The online version contains supplementary material available at 10.1186/s13620-025-00300-8.

## Background

Internationally, bulk tank somatic cell count (BTSCC) is of one of the most important quality parameters of dairy milk production. A BTSCC under 200,000 cells/ml at herd-level is the desired target [1] and is also the typical cow-level threshold used to identify subclinical mastitis [2]. European Law [3] sets the maximum allowable BTSCC for manufacturing milk at 400,000 cells/mL (geometric average over three months, with at least one test conducted per month). Irish legislation dictates that deliveries of milk from a holding that is unable to reduce their geometric mean BTSCC from ≥ 400,000 cells/mL for six consecutive months must be suspended from collection [4]. High BTSCC (> 200,000 cells/ml) indicates either a certain underlying level of subclinical mastitis within the herd [5], or high SCC milk from undiagnosed clinical mastitis cases entering the bulk tank [6]. This affects overall farm income, with Geary et al. [7] reporting that net farm profit decreased as BTSCC increased. Coupled with the finances involved in solving mastitis problems [8] and the fact that mastitis is considered a painful disease with impacts on animal welfare [9], it is imperative for both farmers and livestock that risk factors for increased BTSCC are identified and avoided in everyday dairy practice.

Researchers have demonstrated relationships between farm management practices and BTSCC in international literature. Maintaining cleanliness of farms, houses and milking parlours (‘cleanliness’ assessed using a standardised visual assessment) was strongly associated with lower logarithmic BTSCC in an Irish study by Kelly et al. [10]. These findings align with those of Barkema et al. [11] and Chassagne et al. [12]. Maintenance of farm records is another important aspect of farm management. Hutton et al. [13] reported that farmers in Washington State who were more aware of their herd mastitis status had a lower BTSCC, notably those who managed their records via computer. Hutton et al. [13] also reported that milking clinically infected cows at the end of milking was a practice more commonplace in low SCC herds. Though some studies have investigated the effects of herd size and calving patterns on BTSCC [14–16], there are few which have examined these in conjunction with other specific management practices.

In the parlour, Hutton et al. [13] reported that automatic cluster removers (ACRs) were present less frequently on high SCC farms than low SCC farms. Wenz et al. [17] also documented that the use of ACRs was associated with lower BTSCC on US farms. A study by Baumberger et al. [18] found comparable efficacy between a semi-automated teat scrubber using chlorine dioxide and a conventional pre-dipping routine using 0.5% iodine on reducing teat skin bacterial counts after pre-milking preparation. A UK study by Olde Riekerink et al. [19] showed that BTSCC and individual cow SCC decreased noticeably in farms that implemented an automated post-milking teat dipping and backflushing system compared to their previously utilised spray method of post-milking teat disinfection (PMTD). There is currently limited research available on the effects of the presence of certain parlour additions on BTSCC, particularly those pertaining to cow positioning and milking efficiency.

Pre-milking teat and udder preparation has been found to have no association with BTSCC [10, 13] nor a significant association with lower BTSCC in some studies [20]. However, Galton et al. [21] demonstrated an association between drying of teats and reduced incidence of new intramammary infection (IMI) on American farms. A Canadian study by Goldberg et al. [22] concluded that insufficient hygiene of udders prior to milking may repress the effect of improved management practices. Fore-milking is a pre-milking management practice which is both considered an effective stimulus for milk let-down [23], and an opportunity for farmers to identify any visible abnormalities in the milk, such as blood or clots, indicating clinical mastitis which could contribute to increased BTSCC [24]. Inadequate pre-milking stimulation can cause bimodal milk flow patterns which, though not proven to cause increased BTSCC, can cause machine-induced changes to teat tissue [25–27]. These changes have been linked to increased odds of microbial colonisation of the teat canal [28] and subsequent IMI [29], deteriorating udder health [30], and general discomfort during milking [31]. PMTD is one of the fundamental pillars of the Five-Point Mastitis Control Plan [32] and is crucial in the control of contagious mastitis pathogens [33]. Barkema et al. [11] found the use of teat disinfection reduced BTSCC and Chassagne et al. [12] showed that teat disinfection predominated in low SCC farms compared to high SCC farms. There are few studies which investigate the associations of different combinations of pre- and post-milking practices on BTSCC.

Culling the highest SCC cows until the bulk tank threshold is met has been used as a means of reducing overall BTSCC [34], as well as excluding milk from these cows from the bulk tank collection. Balaine et al. [35] found that lower BTSCC was observed in herds that practiced milk recording; possibly due to increased farmer knowledge on the status of individual cows and more efficient ability to remove those contributing to a high BTSCC. Dry cow management has also been linked with BTSCC. Wenz et al. [17] demonstrated a relationship between antibiotic dry cow therapy (DCT) and low BTSCC. The link between DCT and lower SCC can be attributed to increased cure rates and decreased dry period new IMI [36]. Failure to administer antibiotic-based DCT for high SCC cows was reported by Niemi et al. [37] to have a negative effect on subsequent lactation milk yield and individual cow SCC. A study by Clabby et al. [38] showed that cows treated with internal teat sealant (ITS) alone compared to antibiotics plus ITS had a higher individual logarithmic SCC in the following lactation as well as a higher likelihood of contracting an IMI at calving. There is further scope for investigating the factors which contribute to dry cow management decisions and the effect of frequency of milk recording on BTSCC.

Lastly, individual farmer behaviours, attitudes and beliefs have been shown in literature to impact their farms’ BTSCC. Tarabla & Dodd [39] carried out a survey and reported that the variables related to farmers’ attitude and socio-demographic profile (e.g., positive attitude towards milking, personal values, working within a family unit and being independent) explained a similar or greater amount of the farm performance variation than the management variables did. This is supported by a survey of Dutch dairy farms where farmers’ attitudes and behaviour explained variation in the incidence of mastitis and BTSCC [40]. Schewe et al. [41] conducted a survey of dairy farmers in the eastern USA and also established that farmer beliefs and attitudes regarding mastitis control, antimicrobial use, and labour management could be associated with BTSCC. Kiiman et al. [42] concluded that the milking operator was one of the biggest factors affecting SCC on Estonian dairy farms. Rodrigues et al. [43] found that herds recording higher BTSCC reported less frequent consultation with their herd veterinarian than those with a lower BTSCC. However, they also showed that BTSCC values can rapidly respond when interventions are applied.

While individual components of most dairy management practices have been extensively examined, such investigations frequently occur within controlled experimental settings; not necessarily mirroring the dynamics of operational dairy farms. Few studies directly juxtapose the real-world practices reported by commercial dairy farms solely through the lens of BTSCC metrics. Capturing the complexity and variability inherent in dairy farming may offer some valuable context and applicability to real-life scenarios. Investigation of these from the perspective of a cohort of commercial dairy herds, including investigation of individual farmer beliefs and attitudes towards certain management practices and legislative changes, has not been accomplished in Irish literature. The objective of this paper is to use existing datasets to model the effects of on-farm factors on BTSCC. These factors comprise of farm management practices, parlour management practices and parlour technologies, milking management practices, SCC control strategies, and farmer demographics.

## Materials and methods

### Data

This paper used a previously described dataset which resulted from a farm management and technology survey of 376 commercial Irish dairy farmers in 2022 [44]. Communications were made via phone and email with members of major Irish milk processors (Arrabawn, Aurivo, Bandon, Barryroe, Clonakilty, Centenary Thurles, Dairygold, Drinagh, Tirlán, Lakeland, Limerick Liquid Milk Producers, Lisavaird, Mullinahone, Kerry, Tipperary). The survey link was circulated to all of their suppliers, assumed to be the owners of the herd, by text message. The survey was circulated in July 2022 and farmers were given two months to respond. Mandatory consent was obtained from survey respondents with regard to sharing herd milk recording, bulk milk and stock data from the Irish Cattle Breeding Federation (ICBF) database, and this data was acquired for January 2021 to August 2022. A summary of monthly average farm bulk milk production data for 12 months of 2021, 8 months of 2022 and combined 20 months can be observed in Table A1.

The circulation population considered for the survey were approximately 15,300 specialist dairy farms across 26 counties in the Republic of Ireland [45], with 666 farmers responding to the survey (response rate of 4.35%). Of this, 432 respondents fully completed the survey (64.7%); the remainder submitted surveys which were partially completed. Of the 432 completed responses, 34 farms were removed due to inadequate herd number or contact information in their survey response preventing the extraction of their information on ICBF, 14 were removed as there was no processor data provided to correspond to their survey answers, and one was removed due to inappropriately answered survey questions. Two farms responded to the survey twice. The most recent response for each of these herd numbers was taken as the final response. A total of 376 herds in the dataset supplied milk for 2021 and 381 supplied milk in 2022. Only herds present in the dataset across both years were included in the final analysis, hence, our final dataset contained 376 herds (7,090 monthly observations). A total of 13.0% of respondent herds were removed from the survey dataset during this processing step. The geographical distribution of responses in this study aligns with the distribution of milk producers in Ireland [44].

The survey was divided into five different sections pertaining to: (1) farm-specific management (2), parlour-specific management (including parlour technologies) (3), milking management (4), SCC control strategies, and (5) farmer-specific questions. Section 1 included information on parlour type, parlour manufacturer, mastitis treatment records, the number of cows milking in 2021 and 2022, the frequency of milking, and the number of cows culled in 2021 specifically for high SCC. Section 2 included questions regarding the normal morning and evening milking start times and durations, the number of people involved in milking during peak lactation and the relation of these people to the farmer answering the survey, the age and characteristics of the milking system, information on parlour technological add-ons, frequency of parlour servicing and liner changes, and cluster disinfection practices. Section 3 and Section 4 contained information on fore-milking, pre- and post-milking management, teat disinfection products, glove-wearing practices, California mastitis testing (CMT), and antibiotic and teat sealant application during the 2021 dry-off season. Section 5 included questions about the gender, age, level of education, and years spent in the dairy industry of farmers responding to the survey, as well as an insight into their personal feelings towards udder health management, who they obtain SCC advice from, their attitudes towards the changing legislation on antibiotic usage at dry-off, and their overall satisfaction with their career in dairy.

A full list of survey questions and their relative responses can be found in the Supplementary Materials of Uí Chearbhaill et al. [44]. A condensed list of survey questions and their responses can be found in the Supplementary Materials of this manuscript.

### Data processing

Data were processed using SAS OnDemand for Academics (https://welcome.oda.sas.com/). Herds were identified as seasonal calving, split calving or ‘other’ as per O’Connell et al. (2015). Seasonal calving herds were defined as herds which calved all cows between February and April with peak milk production occurring in May or June and exceeded the herd’s minimum monthly milk production in the herd year by > 700%. Split calving herds supplied milk throughout December and January and had peak milk production that exceeded herd minimum milk production for any month by < 300%. Any herds that did not meet either of these requirements were classified as ‘other’. These considerations resulted in 309 herds being characterised as a seasonal calving system, 29 herds as a split calving system and 38 herds as ‘other’.

Monthly BTSCC and milk volume were logarithmic-10 (log_10_) transformed due to the non-normal distribution of these data, determined through visual analysis of histograms using the PROC UNIVARIATE procedure (SAS OnDemand).

For analysis in this paper, all multiple choice, checkbox, ranking/rating, and dropdown survey answers were coded into a categorical scale for use in the following models.

Efforts were made wherever possible to condense variables for ease of analysis without affecting the significance of the variable. Culling practices (i.e., age, behaviour, fertility, lameness, poor milk production, recurrent incidences of clinical mastitis or persistently high cow-level SCC), previously a ranked ‘1–7’ scale in terms of which were the most prevalent reasons for culling to the least prevalent reasons, were combined into one variable to only reflect the most prevalent reason (i.e., score of ‘1’). The variable regarding who conducts milking on the farm was restructured to account for each individual checkbox answer (i.e., respondents’ self, respondents’ family, or employees of respondents conducting milking) as well as all possible combinations of these. All categories (individual categories and combinations) were mutually exclusive. Fore-milking practices were combined into one variable with 11 mutually exclusive categories reflecting never fore-milking, fore-milking as part of a milking routine (i.e., condensed from the answers of ‘every milking’ and ‘every morning milking’ from the original survey), fore-milking only on suspicion of clinical mastitis (i.e., condensed from the answers of ‘clinically abnormal udders’ and ‘clots in the milk filter’ from the original survey), fore-milking on suspicion of subclinical mastitis (i.e., the answer of ‘increase in BTSCC’ from the original survey) and combinations of any/all of the above. Pre-milking practices included: no pre-milking preparation, a wash step (i.e., condensed from the answers of ‘use of a hose’, ‘washing using an individual udder cloth’ and ‘washing using a communal udder cloth’ from the original survey), a drying step (i.e., condensed from the answers of ‘dry wipe’, and use of ‘individual’ or ‘communal’ cloths used to dry after a washing or disinfecting step from the original survey), a disinfection step (i.e., condensed from the answers of ‘pre-spraying’ or ‘pre-dipping’ from the original survey), and a mechanical intervention (i.e., condensed from the answers of ‘pre-milking wash cup’ and ‘udder brush’ from the original survey). These were combined into a variable reflecting all relevant combinations of these practices, resulting in nine mutually exclusive categories. Udder hygiene practices were combined into one variable with six mutually exclusive categories: clipping tails, clipping udders, flaming udders, as well as combinations of any/all of the above. Storing of mastitis treatment records was combined into a variable with six categories to reflect the use of a whiteboard, a farm recording book, an app, as well as combinations of any/all of the above. Decisions to use teat sealant alone at drying-off were combined into a variable with 21 mutually exclusive categories including: not using any of the listed resources in decisions surrounding selective dry cow therapy, using records of clinical cases throughout lactation, using milk yield records, using cow factors (i.e., age, teat condition, temperament), CMT testing, individual cow records (e.g., individual cow SCC), and combinations of any/all of the above. Sources of advice for SCC control were combined into one variable with 14 mutually exclusive categories including: consulting veterinary professionals, consulting advisory services (i.e., co-op milk quality advisor, other advisor, or on-site visit by specialist mastitis management experts), peer to peer communication (i.e., discussion with colleagues/other farmers), self-directed learning (i.e., use of magazines, websites, or handbooks), and combinations of any/all of the above.

The ordinal categorical variables in the farmer demographics section regarding satisfaction with BTSCC in 2021, satisfaction with BTSCC in 2022, how much respondents believed the new legislation on antibiotic use would affect their current dry-off procedures, how confident respondents felt in being able to maintain their BTSCC using selective dry cow therapy, and how content they were in their dairy careers, (all answers originally in a scale of 1–10), were condensed to reflect answers of ‘1–4’, ‘5–7’ and ‘8–10’ out of a possible ’10’ for ease of analysis. The answers to how much respondents believe a low SCC is achievable on their farm and how useful they find milk recording reports to manage SCC, that were rated ‘1–3’ out of a possible ‘5’, were combined into a ‘≤ 3’ out of ‘5’ variable to account for low response numbers to their individual ranks.

Any answers with only one response have been removed from analysis due to lack of variance. All answers with two responses have been checked for variance in order to determine whether or not they should be removed from the model. Any two response answers with variance similar to other answers for a particular question remained in the model.

### Data analysis

Initially, a multivariable model including all variables across the five different survey sections was considered to investigate the associations of all possible management and farmer demographic factors with BTSCC concurrently. However, the resulting model did not appropriately reflect the nuance within the five survey sections, likely due to non-biologically relevant collinearities between variables from different sections, leading to a degree of overfitting of the model and reduced number of significant variables in the final model. This was deemed to be less impactful and informative than the results obtained from models originating from variables divided into their respective survey sections. However, it is important to note that this five-model structure has its own limitations in that confounding across domains cannot be fully eliminated. Investigations were conducted to establish whether correlations existed between predictor variables. The PROC CORR procedure (SAS OnDemand) was conducted to investigate correlations between ordinal categorical variables using a specified Spearman’s correlation coefficient scale [46] (Table A2). Only very low or low correlations (i.e., *ρ* < 0.2) were found between predictor variables from the survey data. Chi-square tests using PROC FREQ (SAS OnDemand) were conducted between all nominal categorical variables, and between ordinal categorical variables and nominal categorical variables, using a Cramer’s V correlation scale [47] (Table A3). No significant correlations were found between predictor variables. To investigate the relationships between nominal categorical variables and continuous variables, point biserial tests were conducted when the categorical variable had 2 levels and ANOVAs were conducted when there were ≥ 3 levels. Where results were not statistically significant (*p*= > 0.05), no evidence of an association was found. In cases where significance was detected, low R^2^ values (e.g., < 0.2) indicated weak practical relationships. The PROC CORR procedure (SAS OnDemand) was conducted to investigate correlations between continuous variables using a Pearson’s correlation coefficient scale [48] (Table A4). A very strong correlation (*r* = 0.91) was found between ‘morning/AM milking duration’ and ‘evening/PM milking duration’. As a result, two farm management models were created to contain one continuous variable each, and only the stronger model (based on AIC values) was considered for further analysis. The stronger model in this case included the ‘AM milking duration’ continuous variable.

Therefore, five different mixed models were developed to investigate the association between monthly mean Log10BTSCC (log10BTSCC) with the factors in each of the five survey sections. The model building was carried out in a two-step process. Variables remained eligible in the first iteration of the following univariate model (Eq. 1) if they had a level of significance of less than or equal to 10%; i.e., *p* = ≤ 0.1. The second step involved multivariable model building using backwards stepwise elimination. Variables remained in the model if their level of significance was less than 5%; i.e., *p* = < 0.05.

### Model building – Step 1; Univariate analysis

The general base model structure is outlined in Eq. 1:

1 $$\begin{aligned} \log10\mathrm{BTSCC}\;=&\;\mathrm{MONTH}\;+\;\mathrm{YEAR}\;\\&+\;\log10\mathrm{MILKLITRES}\;+\;\mathrm y; \end{aligned}$$

where month (MONTH; categorical variable; January to December of 2021 and January to August of 2022), year (YEAR; categorical variable; 2021 and 2022) and log_10_ monthly milk volume in litres (log10MILKLITRES; continuous variable) were covariates in all five base models as fixed effects and ‘y’ refers to any one of the variables listed in the Supplementary Materials. Herd was included in all models as a repeated measure with a first-order autoregressive covariance structure.

Survey response variables were individually introduced into the base model to establish their univariate association with log10BTSCC. Variables with a *p*-value of ≤ 0.1 remained for further multivariable analysis.

#### Farm-specific management

Five independent farm management variables (41.7%; 5/12) were excluded from input to both of the step two multivariable models due to *p*-values > 0.1. The variable for herd size quartiles was also removed from the model due to its observed relationship with log10milklitres. These excluded variables can be observed in Supplementary Materials 1b.

#### Parlour-specific management and parlour technologies

Twenty-five independent variables (67.6%; 25/37) were excluded from input into the initial mixed model due to *p* values > 0.1. Parlour manufacturer and pulsation were also removed from the model due to multiple implausibilities in survey responses; e.g., pulsation type assigned to a parlour manufacturer that does not produce that type of pulsation. These excluded variables can be observed in Supplementary Materials 2b.

#### Milking management

Four independent variables (50.0%; 4/8) were excluded from inclusion in the initial model due to *p*-values > 0.1. These excluded variables can be observed in Supplementary Materials 3b.

#### SCC control

A variable corresponding to the number of milk recordings conducted in 2021 was added to the model using milk recording data provided from ICBF. Two independent variables (40.0%; 2/5) were excluded from inclusion in the initial model due to *p*-values > 0.1. These excluded variables can be observed in Supplementary Materials 4b.

#### Farmer demographics

Three independent variables (21.4%; 3/14) were excluded from inclusion in the initial model due to *p*-values > 0.1. Education was removed from the model due to the sensitive nature of the question and its lack of demonstrated association with log10BTSCC. The targeted advisory service on animal health (TASAH) dry cow consultation variable was also removed as this service is only available to farmers who have a twelve-month average BTSCC of < 200,000 cells/ml and have also conducted a minimum of four whole herd milk recordings over the same timeframe. All excluded variables can be observed in Supplementary Materials 5b.

### Model building – Step 2; building initial models

An initial multivariable model for each survey section was comprised from all eligible variables identified from step one. Variables with the highest p-value in each iteration of the model were removed until all remaining *p*-values were < 0.05. Each iteration of the model was checked for collinearity by ensuring a difference of ≤ 25% between least square mean estimates for each variable remaining in the model after each iteration. No evidence for collinearity was found.

#### Farm-specific management initial model

The variables included in the initial farm management model were as follows (Supplementary Materials 1a; Table A5):

2 $$\begin{aligned} \log10\mathrm{BTSCC}\;=&\;\mathrm{MONTH}\;+\;\mathrm{YEAR}\;\\&+\;\log10\mathrm{MILKLITRES}\;\\&+\;\mathrm{CALVINGPATTERN}\;\\&+\;\mathrm{MILKER}\;\\&+\;\mathrm{MILKINGFREQUENCY}\;\\&+\;\mathrm{AMDURATION}\;\\&+\;\mathrm{AGEMILKINGSYSTEM}\;\\&+\;\mathrm{CULLING}\;\\&+\;\mathrm{MASTITISTXRECORDS}, \end{aligned}$$

where CALVINGPATTERN = calving pattern (categorical variable; seasonal, split, other), MILKER = person who conducts the milking (categorical variable; respondents’ self, family of respondent, employee of respondent, combination of any/all of the above), MILKINGFREQUENCY = the frequency of milking (categorical variable; once per day, twice per day, automated milking system), AMDURATION = duration of morning milkings (continuous variable; minutes), AGEMILKINGSYSTEM = the age of the milking system (categorical variable; <5 years to 36 + years in five year increments), CULLING = primary reasons for culling on respondent farms (categorical variable; age, behaviour, fertility, lameness, poor milk production, recurrent incidences of clinical mastitis, persistently high SCC), MASTITISTXRECORDS = keeping of mastitis treatment records (categorical variable; yes/no), and herd is included as a repeated effect.

#### Parlour-specific management and parlour technologies initial model

The variables included in the initial parlour management and technology model were as follows (Supplementary Materials 2a; Table A6):

3 $$\begin{aligned} \log10\mathrm{BTSCC}\;=&\;\mathrm{MONTH}\;+\;\mathrm{YEAR}\;\\&+\;\log10\mathrm{MILKLITRES}\;\\&+\;\mathrm{PARLOURTYPE}\;\\&+\;\mathrm{STRAIGHTBRRAIL}\;\\&+\;\mathrm{MANBAILING}\;\\&+\;\mathrm{NOADDONS}\;\\&+\;\mathrm{AUTOCLUSREM}\;\\&+\;\mathrm{AUTOWASHMM}\;\\&+\;\mathrm{BACKINGGATE}\;\\&+\;\mathrm{VARSPEEDVACC}\;\\&+\;\mathrm{LINERCHANGE}\;\\&+\;\mathrm{CLUSTERDISINFECT}, \end{aligned}$$

where PARLOURTYPE = parlour type (categorical variable; swing-over herringbone, double-up herringbone, herringbone with recording jars, parallel, rotary, automated milking system (AMS), other), STRAIGHTBRRAIL = straight breast rails in the parlour as a means of positioning cows for milking (categorical variable; yes/no), MANBAILING = manual bailing systems to assist with cow positioning (categorical variable; yes/no), NOADDONS = no technology add-ons in the parlour (categorical variable; yes/no), AUTOCLUSREM = technology add-ons of automatic cluster removers (categorical variable; yes/no), AUTOWASHMM = automatic washers on the milking machine (categorical variable; yes/no), BACKINGGATES = backing gates in the collecting yard (categorical variable; yes/no), VARSPEEDVACC = variable speed vacuum pumps (categorical variable; yes/no), LINERCHANGE = frequency of liner changes (categorical variable; once per year, twice per year, three times per year, every 2 years, every 2000 milkings, every 2500 milkings, other), CLUSTERDISINFECT = whether or not clusters are disinfected during milking (categorical variable; yes/no), and herd is included as a repeated effect.

#### Milking management initial model

The variables included in the initial milking management model were as follows (Supplementary Materials 3a; Table A7):

4 $$\begin{aligned} \mathbf{log}10\mathrm{BTSCC}\;=&\;\mathrm{MONTH}\;+\;\mathrm{YEAR}\;\\&+\;\log10\mathrm{MILKLITRES}\;\\&+\;\mathrm{FOREMILK}\;\\&+\;\mathrm{PREMILK}\;\\&+\;\mathrm{POSTMILK}\;\;\\&+\;\;\mathrm{UDDERHYGIENE}, \end{aligned}$$

where FOREMILK = when, if ever, respondents conduct fore-milking (categorical variable; never, as part of milking routine, for clinical mastitis indications, for subclinical mastitis indications, after calving, combinations of any/all of the above), PREMILK = pre-milking udder preparation practices (categorical variable; none, wash step, drying step, disinfection step, mechanical intervention, combinations of any/all of the above), POSTMILK = use of post-milking teat disinfection (categorical variable; none, spraying, dipping, automatic in-cluster dipping), UDDERHYGIENE = udder hygiene practices (categorical variable; clip tails, clip udders, flame udders, combination of any/all of the above), and herd is included as a repeated effect.

#### SCC control initial model

The variables included in the initial SCC control model were as follows (Supplementary Materials 4a; Table A8):

5 $$\begin{aligned} \log10\mathrm{BTSCC}\;=&\;\mathrm{MONTH}\;+\;\mathrm{YEAR}\;\\&+\;\log10\mathrm{MILKLITRES}\;\\&+\;\mathrm{RECORDSKEPT}\;\\&+\;\mathrm{TSONLY}\;\\&+\;\mathrm{MILKRECORDINGS}, \end{aligned}$$

where RECORDSKEPT = where respondents keep their mastitis treatment records (categorical variable; whiteboard, farm recording book, app, combination of any/all of the above), TSONLY = decisions used for selective dry cow therapy (categorical variable; none of the listed resources, records of clinical cases and their outcomes throughout lactation, milk yield records, cow factors, CMT testing, individual cow records, combinations of any/all of the above), MILKRECORDINGS = the number of milk recordings conducted in 2021 (categorical variable; 0–11), and herd is included as a repeated effect.

#### Farmer demographics initial model

The variables included in the initial farmer demographic model were as follows (Supplementary Materials 5a; Table A9):

6 $$\begin{aligned} \log10\mathrm{BTSCC}\;=&\;\mathrm{MONTH}\;+\;\mathrm{YEAR}\;\\&+\;\log10\mathrm{MILKLITRES}\;\\&+\;\mathrm{YEARSDAIRYING}\;\\&+\;\mathrm{SCC}2021\;\\&+\;\mathrm{SCC}2022\;\\&+\;\mathrm{LOWSCCACHIEVEABLE}\;\\&+\;\mathrm{REPORTUSEFULNESS}\;\\&+\;\mathrm{SCCADVICE}\;\\&+\;\mathrm{HIGHSCCFROM}\;\\&+\;\mathrm{LEGISLATIONEFFECT}\;\\&+\;\mathrm{CONFIDENCESDCT}, \end{aligned}$$

where YEARSDAIRYING = years spent in the dairy profession (categorical variable; <5 years, 5–10 years, 10–20 years, 20–30 years, 30–40 years, 40 + years), SCC2021 = the level of satisfaction with overall BTSCC in 2021 (categorical variable; ‘1–4/10’, ‘5–7/10’, ‘8–10/10’), SCC2022 = the level of satisfaction with overall BTSCC up until August of 2022 (categorical variable; ‘1–4/10’, ‘5–7/10’, ‘8–10/10’), LOWSCCACHIEVABLE = whether or not respondents believe a low SCC (< 200,000 cells/ml) is achievable on their farm (categorical variable;'≤ 3/5','4/5','5/5'), REPORTUSEFULNESS = how useful respondents find milk recording reports in reducing BTSCC (categorical variable;'≤ 3/5','4/5','5/5'), SCCADVICE = where respondents source advice on SCC (categorical variable; veterinary professional, advisory services, peer to peer communication, self-directed learning, combinations of any/all of the above), HIGHSCCFROM = what respondents believe is the greatest cause of high SCC on dairy farms (categorical variable; milking machine, milking process/practices, freshly calved cows, older cows, housing, grassland, unknown source, other), LESISLATIONEFFECT = the degree to which the new legislation on antibiotic use affect respondents’ current antibiotic usage at dry-off (categorical variable; ‘1–4/10’, ‘5–7/10’, ‘8–10/10’), CONFIDENCESDCT = respondent confidence in their ability to manage their BTSCC with selective dry cow therapy (categorical variable; ‘1–4/10’, ‘5–7/10’, ‘8–10/10’), and herd is included as a repeated effect.

## Results

Not all models have the same number of responses. This is due to variety in the number of survey response answers per question depending on the section addressed [44]. The magnitude of the significance is not equitable across all variables. Strength of significance can be determined by estimates observable in each of the multivariable linear regression tables (Tables 2, 4, 6, 8 and 10).

### Farm-specific management

The significant variables (*p* = < 0.05) remaining in the final farm management model can be observed in Table 1.

**Table 1:** Type 3 estimates of fixed effects of variables in the farm-specific management model significantly associated with BTSCC (*n* = 338 respondents)

Effect	F value	*p*-value
MONTH	47.75	< 0.0001
YEAR	2.61	0.11
log10MILKLITRES	43.62	< 0.0001
CALVINGPATTERN	10.50	< 0.0001
MILKER	2.25	0.038
MASTITISTXRECORDS	7.88	0.0053
AMDURATION	8.48	0.0038

MONTH = month of the year (categorical variable; January to December for 2021 and January to August of 2022), YEAR = year (categorical variable; 2021 or 2022), log10MILKLITRES = log10 of the monthly bulk tank milk yield (continuous variable; litres), CALVINGPATTERN = calving pattern (categorical variable; seasonal, split, other), MILKER = who conducts the milking on the farm (categorical variable; respondent conducts milking themselves, family of the respondent conducts milking, employee of the respondent conducts milking, combinations of any/all of the above), AMDURATION = duration of morning milking (continuous variable; minutes)

Associations between these farm management variables and log10BTSCC can be observed in Table 2. Seasonal calving systems were associated with significantly lower log10BTSCC than either split or ‘other’ calving systems (*p* = 0.0061 and *p* = 0.0001, respectively). Employees conducting the milking alone or in conjunction with the respondent was associated with a significant increase in log10BTSCC (*p* = 0.029 and *p* = 0.011, respectively; Supplementary Table 1) compared to respondents and their family milking together. Respondents milking with a family member was also associated with a significant decrease in log10BTSCC compared to respondents milking alone (*p* = 0.027; Supplementary Table 1), or family members milking alone (*p* = 0.042; Supplementary Table 1). The keeping of mastitis treatment records was also associated with a significant decrease in log10BTSCC (*p* = 0.0053). Longer duration of morning milking was significantly associated with an increased log10BTSCC (*p* = 0.0038), though the effect size was particularly low (0.00074).

**Table 2:** Multivariable logistic regression model showing associations between BTSCC and aspects of farm-specific management for the significant main effects (*n* = 338 respondents)

Effect		Estimate	Standard Error	*p*-value	LS means estimate	*n*
Intercept		5.41	0.038	< 0.0001	.	338
MONTH	January	Reference	.	.	5.35	338
	February	−0.028	0.0075	0.0002	5.32	338
	March	−0.14	0.011	< 0.0001	5.21	338
	April	−0.19	0.012	< 0.0001	5.16	338
	May	−0.18	0.013	< 0.0001	5.17	338
	June	−0.16	0.013	< 0.0001	5.19	338
	July	−0.12	0.013	< 0.0001	5.23	338
	August	−0.10	0.013	< 0.0001	5.25	338
	September	−0.10	0.014	< 0.0001	5.25	338
	October	−0.059	0.014	< 0.0001	5.29	338
	November	−0.022	0.013	0.099	5.33	338
	December	0.015	0.013	0.23	5.37	338
YEAR	2021	Reference	.	.	5.27	338
	2022	−0.018	0.011	0.11	5.25	338
log10MILKLITRES	Litres	−0.050	0.0075	< 0.0001	.	338
CALVINGPATTERN	Seasonal	Reference	.	.	5.20	283
	Split	0.080	0.029	0.0061	5.28	23
	Other	0.097	0.025	0.0001	5.30	32
MILKER	Respondent conducts milking themselves	Reference	.	.	5.25	79
	Family of respondent conducts milking	0.028	0.036	0.44	5.28	17
	Employee(s) of respondent conducts milking	0.060	0.048	0.21	5.31	9
	Respondent and family of respondent conduct milking	−0.043	0.019	0.027	5.21	129
	Respondent and employee(s) of respondent conduct milking	0.016	0.025	0.53	5.27	51
	Family and employee(s) of respondent conduct milking	0.018	0.062	0.77	5.27	5
	Respondent, family of respondent and employee(s) of respondent conduct milking	−0.015	0.025	0.55	5.24	48
MASTITISTXRECRODS	No; I do not keep mastitis treatment records	Reference	.	.	5.29	305
	Yes; I keep mastitis treatment records	−0.069	0.025	0.0053	5.23	33
AMDURATION	Minutes	0.00074	0.00025	0.0038	.	338

### Parlour-specific management and parlour technologies

The significant variables (*p* = < 0.05) which remained in the final parlour management and technologies model can be observed in Table 3.

**Table 3:** Type 3 estimates of fixed effects of variables in the parlour-specific management and parlour technologies model significantly associated with BTSCC (*n* = 354 respondents)

Effect	F value	*p*-value
MONTH	50.51	< 0.0001
YEAR	1.34	0.25
log10MILKLITRES	34.24	< 0.0001
PARLOURTYPE	4.01	0.0007
STRAIGHTBRRAIL	5.23	0.023
AUTOCLUSREM	3.96	0.047
AUTOWASHMM	6.89	0.0090
BACKINGGATES	5.32	0.022
CLUSTERDISINFECT	6.14	0.014

MONTH = month of the year (categorical variable; January to December for 2021 and January to August of 2022), YEAR = year (categorical variable; 2021 or 2022), log10MILKLITRES = log10 of the monthly bulk tank milk yield (continuous variable; litres), PARLOURTYPE = parlour type (categorical variable; yes/no), STRAIGHTBRRAIL = straight breast rails (categorical variable; yes/no), AUTOCLUSREM = automatic cluster removers (categorical variable; yes/no), AUTOWASHMM = automatic washer on the milking machine (categorical variable; yes/no), BACKINGGATES = backing gates in the collecting yard (categorical variable; yes/no), CLUSTERDISINFECT = whether cluster disinfection is carried out on respondent farms (categorical variable; yes/no)

Associations between these parlour management and technology variables and log10BTSCC can be observed in Table 4. Herringbone parlours with recording jars had a significantly lower log10BTSCC than swing-over herringbones (*p* = 0.0028; Supplementary Table 2). Conversely, rotary parlours had a significantly higher log10BTSCC than swing-over herringbones (*p* = 0.011; Supplementary Table 2), herringbones with recording jars (*p* = 0.0006; Supplementary Table 2) and parallel parlours (*p* = 0.0075; Supplementary Table 2). In terms of cow positioning, the presence of straight breast rails in a parlour was associated with significantly increased log10BTSCC than for parlours without them (*p* = 0.023). The presence of ACRs and automatic washers on the milking machine were associated with significant decreases in log10BTSCC compared to parlours which did not have either (*p* = 0.047 and *p* = 0.009, respectively). In contrast, the presence of backing gates in the collecting yard was associated with a significant increase in log10BTSCC compared to farms without backing gates (*p* = 0.022). Cluster disinfection practices being carried out on farm was associated with a significant decrease in log10BTSCC compared to farms which do not disinfect their clusters (*p* = 0.014).

**Table 4:** Multivariable logistic regression model showing associations between BTSCC and aspects of parlour-specific management and parlour technologies for the significant main effects (*n* = 354 respondents)

Effect		Estimate	Standard Error	*p*-value	LS means estimate	*n*
Intercept		5.60	0.069	<0.0001	.	354
MONTH	January	Reference	.	.	5.34	354
	February	-0.030	0.0071	<0.0001	5.31	354
	March	-0.14	0.010	<0.0001	5.20	354
	April	-0.19	0.012	<0.0001	5.14	354
	May	-0.18	0.012	<0.0001	5.16	354
	June	-0.16	0.012	<0.0001	5.17	354
	July	-0.12	0.012	<0.0001	5.21	354
	August	-0.10	0.012	<0.0001	5.24	354
	September	-0.10	0.013	<0.0001	5.24	354
	October	-0.061	0.013	<0.0001	5.28	354
	November	-0.023	0.013	0.076	5.31	354
	December	0.012	0.012	0.32	5.35	354
YEAR	2021	Reference	.	.	5.25	354
	2022	-0.013	0.011	0.25	5.24	354
log10MILKLITRES	Litres	-0.043	0.0074	<0.0001	.	354
PARLOURTYPE	Swing-over herringbone	Reference	.	.	5.21	214
	Automatic milking system (AMS)	0.092	0.062	0.14	5.31	5
	Double-up herringbone	0.063	0.035	0.069	5.28	17
	Herringbone with recording jars	-0.057	0.019	0.0028	5.16	82
	Parallel	-0.027	0.030	0.36	5.19	24
	Rotary	0.13	0.051	0.011	5.35	8
	Other	0.015	0.068	0.83	5.23	4
STRAIGHTBRRAIL	No straight breast rails	Reference	.	.	5.21	331
	Straight breast rails	0.068	0.030	0.023	5.28	23
AUTOCLUSREM	No automatic cluster removers	Reference	.	.	5.26	149
	Automatic cluster removers	-0.034	0.017	0.047	5.23	205
AUTOWASHMM	No automatic washer on the milking machine	Reference	.	.	5.27	232
	Automatic washer on the milking machine	-0.048	0.018	0.009	5.22	122
BACKINGGATES	No backing gates in the collecting yard	Reference	.	.	5.22	309
	Collecting gates in the collecting yard	0.053	0.023	0.022	5.27	45
CLUSTERDISINFECT	Cluster disinfection; no	Reference	.	.	5.26	234
	Cluster disinfection; yes	-0.039	0.016	0.014	5.23	120

### Milking management

The significant variables (*p* = < 0.05) which remained in the final milking management model can be observed in Table 5.

**Table 5:** Type 3 estimates of fixed effects of variables in the milking management model significantly associated with BTSCC (*n* = 337 respondents)

Effect	F value	*p*-value
MONTH	50.89	< 0.0001
YEAR	2.89	0.090
log10MILKLITRES	29.89	< 0.0001
FOREMILK	1.87	0.049
PREMILK	3.09	0.0023
POSTMILK	2.94	0.033
UDDERHYGIENE	3.86	0.0021

MONTH = month of the year (categorical variable; January to December for 2021 and January to August of 2022), YEAR = year (categorical variable; 2021 or 2022), log10MILKLITRES = log10 of the monthly bulk tank milk yield (continuous variable; litres), FOREMILK = when, if ever, respondents conduct fore-milking (categorical variable; never, as part of a milking routine, for clinical mastitis indications, for subclinical mastitis indications, after calving, combinations of any/all of the above), PREMILK = pre-milking preparation, (categorical variable; never, wash step, drying step, disinfection step, mechanical intervention, combinations of any/all of the above), POSTMILK = post-milking teat disinfection (categorical variable; none, spraying, dipping, automatic in-cluster dipping), UDDERHYGIENE = practices to maintain good udder hygiene (categorical variable; clip tails, clip udders, flame udders, combinations of any/all of the above)

Associations between these milking management variables and log10BTSCC can be observed in Table 6. Never conducting fore-milking was associated with an increased log10BTSCC compared to fore-milking as part of a milking routine (*p* = 0.027; Supplementary Table 3) and fore-milking after calving (*p* = 0.031; Supplementary Table 3). Fore-milking based on a combined suspicion of clinical and subclinical mastitis was associated with a higher log10BTSCC than fore-milking as part of a milking routine (*p* = 0.018; Supplementary Table 3) or fore-milking after calving (*p* = 0.035; Supplementary Table 3). A combination of fore-milking for clinical mastitis indications, subclinical mastitis indications, and after calving also had a significantly higher log10BTSCC than either fore-milking as part of a milking routine (*p* = 0.0020; Supplementary Table 3) or fore-milking after calving (*p* = 0.020; Supplementary Table 3) alone; though the effect size for this combination was particularly low (−0.007). No pre-milking udder preparation was associated with a significant increase in log10BTSCC compared to a combination of a disinfection and drying step (*p* = 0.0004). Disinfecting and drying teats was also associated with a significant decrease in log10BTSCC compared to either a dry or a disinfection step alone (*p* = 0.0017 and p = < 0.0001, respectively; Supplementary Table 3), a combined wash and drying step (*p* = 0.011; Supplementary Table 3), and a combined wash and disinfection step (*p* = 0.0056; Supplementary Table 3). A wash step alone was associated with a significant decrease in log10BTSCC compared to a disinfection step alone (*p* = 0.017; Supplementary Table 3) or a combined wash and disinfection step (*p* = 0.030).

No PMTD was associated with a significant increase in log10BTSCC compared to spraying and in-cluster dipping (*p* = 0.034 and *p* = 0.0072, respectively; Supplementary Table 3). Spraying and dipping were both associated with an increased log10BTSCC compared to automatic in-cluster dipping (*p* = 0.043 and *p* = 0.035, respectively; Supplementary Table 3). A combination of clipping tails and clipping udders for maintenance of good udder hygiene was significantly associated with an increased log10BTSCC compared to flaming udders alone (*p* = 0.023; Supplementary Table 3), clipping tails alone (*p* = 0.0002; Supplementary Table 3), a combination of clipping tails and flaming udders (*p* = 0.0021; Supplementary Table 3), and a combination of clipping tails, clipping udders and flaming udders (*p* = 0.0080; Supplementary Table 3).

**Table 6:** Multivariable logistic regression model showing associations between BTSCC and aspects of milking management for the significant main effects (*n* = 337 respondents)

Effect		Estimate	Standard Error	*p*-value	LS means estimate	*n*
Intercept		5.59	0.077	< 0.0001	.	337
MONTH	January	Reference	.	.	5.22	337
	February	−0.036	0.0073	< 0.0001	5.19	337
	March	−0.15	0.011	< 0.0001	5.07	337
	April	−0.20	0.012	< 0.0001	5.02	337
	May	−0.19	0.013	< 0.0001	5.03	337
	June	−0.17	0.013	< 0.0001	5.05	337
	July	−0.13	0.013	< 0.0001	5.092	337
	August	−0.11	0.013	< 0.0001	5.11	337
	September	−0.11	0.013	< 0.0001	5.11	337
	October	−0.066	0.013	< 0.0001	5.15	337
	November	−0.024	0.013	0.069	5.20	337
	December	0.012	0.013	0.33	5.23	337
YEAR	2021	Reference	.	.	5.13	337
	2022	−0.019	0.011	0.09	5.11	337
log10MILKLITRES	Litres	−0.041	0.0074	< 0.0001	.	337
FOREMILK	Never fore-milk	Reference	.	.	5.16	18
	Fore-milk as part of milking routine	−0.084	0.038	0.027	5.081	65
	Fore-milk for clinical mastitis indications	−0.044	0.038	0.24	5.12	49
	Fore-milk for subclinical mastitis indications	−0.021	0.053	0.69	5.14	11
	Fore-milk after calving	−0.14	0.065	0.031	5.024	7
	Fore-milk as part of milking routine and for clinical mastitis indications	−0.10	0.076	0.18	5.064	4
	Fore-milk for clinical mastitis indications and subclinical mastitis indications	−0.013	0.041	0.75	5.15	34
	Fore-milk for clinical mastitis indications and after calving	−0.039	0.037	0.29	5.13	71
	Fore-milk for subclinical mastitis indications and after calving	0.081	0.086	0.35	5.25	3
	Fore-milk for clinical mastitis indications, subclinical mastitis indications, and after calving	−0.0070	0.037	0.85	5.16	73
	Fore-milk as part of milking routine, for clinical and subclinical mastitis indications, and after calving	−0.085	0.10	0.40	5.089	2
PREMILK	No pre-milking udder preparation	Reference	.	.	5.12	106
	Drying step	−0.0097	0.020	0.63	5.11	97
	Wash step	−0.066	0.041	0.11	5.055	13
	Disinfection step	0.042	0.027	0.12	5.16	36
	Mechanical intervention	0.039	0.068	0.57	5.16	5
	Wash and drying step	−0.0044	0.031	0.89	5.12	26
	Disinfection and drying step	−0.092	0.026	0.0004	5.029	42
	Wash and disinfection step	0.096	0.066	0.14	5.22	5
	Wash step, disinfection step and drying step	0.017	0.054	0.76	5.14	7
POSTMILK	None	Reference	.	.	5.24	10
	Spraying	−0.097	0.045	0.034	5.15	304
	Dipping	−0.077	0.054	0.16	5.17	21
	Automatic in-cluster dipping	−0.31	0.11	0.0072	4.93	2
UDDERHYGIENE	Clip udders	Reference	.	.	5.13	7
	Flame udders	−0.063	0.081	0.44	5.07	5
	Clip tails	0.00018	0.054	1.00	5.13	238
	Clip tails and clip udders	0.084	0.057	0.14	5.22	48
	Clip tails and flame udders	−0.015	0.060	0.80	5.12	32
	Clip tails, clip udders and flame udders	−0.065	0.075	0.39	5.069	7

### SCC control

The significant variables (*p* = < 0.05) which remained in the final SCC control model can be observed in Table 7.

**Table 7:** Type 3 estimates of fixed effects of variables in the SCC control model significantly associated with BTSCC (*n* = 318 respondents)

Effect	F value	*p*-value
MONTH	50.94	< 0.0001
YEAR	3.11	0.079
log10MILKLITRES	12.68	0.0004
TSONLY	2.49	0.0005
MILKRECORDINGS	3.61	< 0.0001

MONTH = month of the year (categorical variable; January to December for 2021 and January to August of 2022), YEAR = year (categorical variable; 2021 or 2022), log10MILKLITRES = log10 of the monthly bulk tank milk yield (continuous variable; litres), TSONLY = what information respondents use to decide on selective dry cow therapy (categorical variable; none of the listed resources, records of clinical cases throughout lactation, milk yield records, cow factors, CMT testing, individual cow records, combinations of any/all of the above), MILKRECORDINGS = the number of milk recordings conducted in 2021 (categorical variable; 0–11)

Associations between these SCC control variables and log10BTSCC can be observed in Table 8. Farms not using any of the listed resources for decisions relating to selective dry cow therapy were associated with a significantly higher log10BTSCC compared to farms that use records of clinical mastitis alone (*p* = 0.026; Supplementary Table 4), and farms that use clinical mastitis records in conjunction with milk yield records (*p* = 0.0079; Supplementary Table 4), cow factors (*p* = 0.0074; Supplementary Table 4), or individual cow records (*p* = 0.0015; Supplementary Table 4). Decisions for selective dry cow therapy made on the basis of all available resources listed was significantly associated with lower log10BTSCC than most other possible combinations. The use of milk recording records alone (i.e., individual cow records), was associated with a significant increase in log10BTSCC compared to using a combination of all available resources (*p* = 0.0040; Supplementary Table 4). Conducting zero milk recordings in 2021 was associated with a significant increase in log10BTSCC compared to conducting one (*p* = 0.013; Supplementary Table 4) or eleven recordings (*p* = 0.025; Supplementary Table 4), but was associated with a significant decrease in log10BTSCC compared to three recordings (*p* = 0.0008; Supplementary Table 4).

**Table 8:** Multivariable logistic regression model showing associations between BTSCC and aspects of SCC control for the significant main effects (*n* = 318 respondents)

Effect		Estimate	Standard Error	*p*-value	LS means estimate	*n*
Intercept		5.47	0.040	< 0.0001	.	318
MONTH	January	Reference	.	.	5.25	318
	February	−0.033	0.0075	< 0.0001	5.22	318
	March	−0.15	0.011	< 0.0001	5.10	318
	April	−0.21	0.012	< 0.0001	5.046	318
	May	−0.19	0.013	< 0.0001	5.058	318
	June	−0.18	0.013	< 0.0001	5.073	318
	July	−0.13	0.013	< 0.0001	5.12	318
	August	−0.11	0.013	< 0.0001	5.14	318
	September	−0.11	0.014	< 0.0001	5.14	318
	October	−0.068	0.014	< 0.0001	5.18	318
	November	−0.030	0.014	0.029	5.22	318
	December	0.0076	0.013	0.55	5.26	318
YEAR	2021	Reference	.	.	5.16	318
	2022	−0.020	0.012	0.079	5.14	318
log10MILKLITRES	Litres	−0.028	0.0078	0.0004	.	318
TSONLY	None of the listed resources	Reference	.	.	5.21	29
	Records of clinical cases throughout lactation	−0.078	0.035	0.026	5.13	29
	Milk yield records	−0.074	0.041	0.074	5.14	16
	Cow factors	0.076	0.053	0.15	5.2	8
	CMT testing	−0.021	0.063	0.73	5.19	5
	Individual cow records	−0.055	0.030	0.069	5.16	67
	Records of clinical cases and milk yield records	−0.13	0.048	0.0079	5.081	10
	Records of clinical cases and cow factors	−0.18	0.069	0.0074	5.026	4
	Records of clinical cases and individual cow records	−0.10	0.032	0.0015	5.11	45
	Milk yield records and cow factors	0.044	0.074	0.55	5.25	4
	Milk yield records and individual cow records	−0.067	0.10	0.51	5.14	2
	Cow factors and individual cow records	−0.046	0.069	0.50	5.16	4
	Records of clinical cases, milk yield records, and cow factors	−0.15	0.059	0.011	5.061	6
	Records of clinical mastitis, milk yield records, and individual cow records	0.010	0.051	0.84	5.22	9
	Records of clinical mastitis, cow factors, and individual cow records	−0.12	0.036	0.0013	5.093	27
	Records of clinical mastitis, CMT testing, and individual cow records	−0.11	0.044	0.01	5.096	13
	Cow factors, CMT testing, and individual cow records	0.012	0.078	0.88	5.22	3
	Records of clinical cases, milk yield records, cow factors, and individual cow records	−0.048	0.051	0.34	5.16	9
	Records of clinical cases, milk yield records, CMT testing, and individual cow records	0.065	0.094	0.49	5.28	2
	Records of clinical cases, cow factors, CMT testing, and individual cow records	−0.092	0.043	0.035	5.12	14
	Records of clinical cases, milk yield records, cow factors, CMT testing, and individual cow records	−0.17	0.045	0.0002	5.038	12
MILKRECORDINGS	0	Reference	.	.	5.17	59
	1	−0.20	0.081	0.013	4.97	3
	2	0.12	0.080	0.13	5.29	3
	3	0.12	0.035	0.0008	5.29	20
	4	0.0024	0.024	0.92	5.17	98
	5	−0.023	0.026	0.38	5.15	64
	6	0.015	0.031	0.64	5.19	31
	7	−0.011	0.033	0.73	5.16	26
	8	−0.092	0.077	0.24	5.08	3
	9	−0.11	0.078	0.17	5.06	3
	10	0.090	0.072	0.21	5.26	4
	11	−0.15	0.067	0.025	5.019	4

### Farmer demographics

The significant variables (*p* = < 0.05) which remained in the final farmer demographics model can be observed in Table 9.

**Table 9:** Type 3 estimates of fixed effects of variables in the farmer demographics model significantly associated with BTSCC (*n* = 363 respondents)

Effect	F value	*p*-value
MONTH	53.75	< 0.0001
YEAR	1.80	0.17
log10MILKLITRES	32.00	< 0.0001
YEARSDAIRYING	4.39	0.0005
SCC2021	60.85	< 0.0001
SCC2022	13.59	< 0.0001
LOWSCCACHIEVABLE	6.28	< 0.0001
HIGHSCCFROM	3.25	0.0014
SCCADVICE	3.25	0.0016

MONTH = month of the year (categorical variable; January to December for 2021 and January to August of 2022), YEAR = year (categorical variable; 2021 or 2022), log10MILKLITRES = log10 of the monthly bulk tank milk yield (continuous variable; litres), YEARSDAIRYING = how many years respondents have spent dairying (categorical variable; <5 years, 5–10 years, 10–20 years, 20–30 years, 30–40 years, 40 + years), SCC2021 = scale of satisfaction with BTSCC in 2021 (categorical variable; ‘1–3/10’, ‘4–7/10’, ‘8–10/10’), SCC2022 = scale of satisfaction with BTSCC in 2022 (categorical variable; ‘1–3/10’, ‘4–7/10’, ‘8–10/10’), LOWSCCACHIEVABLE = scale of whether respondents believe a BTSCC of < 200,000 cells/ml is achievable on their farm (categorical variable;'≤3/5','4/5','5/5'), HIGHSCCFROM = what respondents believe is the main source of high SCC on farms (categorical variable; milking machine, milking process/practices, freshly calved cows, older cows, housing, grassland, unknown source, other), SCCADVICE = where respondents seek advice on SCC from (categorical variable; veterinary professional, advisory services, peer to peer communication, self-directed learning, combinations of any/all of the above)

Associations between these farmer demographics variables and log10BTSCC can be observed in Table 10. Farmers dairying for less than five years were associated with a significantly lower log10BTSCC than those milking more than 5 years (Supplementary Table 5). Those milking 5–10 years showed a significantly lower log10BTSCC compared to those milking for 20–30 years (*p* = 0.017; Supplementary Table 5) and 40 + years (*p* = 0.049; Supplementary Table 5). Regarding their satisfaction with their BTSCC in 2021, respondents who awarded scores of ‘1–4’ had a significantly higher log10BTSCC than those who awarded a score of ‘5–7’ and ‘8–10’ (*p* = < 0.0001; Supplementary Table 5). For 2022, those who awarded scores of ‘8–10’ were associated with a significantly lower log10BTSCC than all other scores (*p* = < 0.0001; Supplementary Table 5). Farmers who believed that a BTSCC of < 200,000 cells/ml was likely not possible on their farm (i.e., ≤ ‘3’ out of ‘5’) were associated with a significantly higher log10BTSCC than those who scored their belief at a ‘4’ or a ‘5’ (*p* = 0.0016 and *p* = < 0.0001, respectively; Supplementary Table 5).

Farmers who believed that milking practices were more responsible for high SCC had a significantly increased log10BTSCC compared to those who believed that housing was the culprit (*p* = 0.0021; Supplementary Table 5), but significantly decreased log10BTSCC compared to those who believed older cows were (*p* = < 0.0001; Supplementary Table 5). With regard to where they seek advice on SCC, consultation with advisory services alone was associated with a significant increase in log10BTSCC compared to advice from veterinary professionals alone (*p* = 0.0013; Supplementary Table 5), from peer to peer communication alone (*p* = 0.0001; Supplementary Table 5), and from self-directed learning alone (*p* = 0.042; Supplementary Table 5). The use of advisory services alone was also associated with a significant increase in log10BTSCC compared to using their advice in conjunction with veterinary professionals (*p* = 0.0013; Supplementary Table 5), with a combination of veterinary professionals and self-directed learning (*p* = < 0.0001; Supplementary Table 5), with a combination of veterinary professionals and peer to peer communication (*p* = < 0.0001; Supplementary Table 5), and with a combination of veterinary professionals, peer to peer communication and self-directed learning (*p* = 0.0035; Supplementary Table 5). Advice from a veterinary professional alone was associated with a significant increase in BTSCC compared to advice obtained from a combination of a veterinary professional, advisory services and peer to peer communication (*p* = 0.036; Supplementary Table 5) and a combination of a veterinary professional, advisory services and self-directed learning (*p* = 0.037; Supplementary Table 5).

**Table 10:** Multivariable logistic regression model showing associations between BTSCC and aspects of farmer demographics for the significant main effects (*n* = 363 respondents)

Effect		Estimate	Standard Error	*p*-value	LS means estimate	*n*
Intercept		5.63	0.055	< 0.0001	.	363
MONTH	January	Reference	.	.	5.39	363
	February	−0.029	0.0070	< 0.0001	5.36	363
	March	−0.14	0.010	< 0.0001	5.24	363
	April	−0.19	0.011	< 0.0001	5.19	363
	May	−0.18	0.012	< 0.0001	5.21	363
	June	−0.17	0.012	< 0.0001	5.22	363
	July	−0.12	0.012	< 0.0001	5.26	363
	August	−0.10	0.012	< 0.0001	5.28	363
	September	−0.10	0.012	< 0.0001	5.29	363
	October	−0.061	0.012	< 0.0001	5.33	363
	November	−0.020	0.012	0.088	5.37	363
	December	0.018	0.011	0.11	5.40	363
YEAR	2021	Reference	.	.	5.30	363
	2022	−0.013	0.0094	0.17	5.29	363
LOG10MILKLITRES	Litres	−0.040	0.0071	< 0.0001	.	363
YEARSDAIRYING	< 5 years	Reference	.	.	5.16	19
	5–10 years	0.069	0.030	0.020	5.23	49
	10–20 years	0.10	0.029	0.0004	5.26	56
	20–30 years	0.11	0.027	< 0.0001	5.27	96
	30–40 years	0.097	0.027	0.0005	5.25	75
	40 + years	0.11	0.028	0.0001	5.27	68
SCC2021	1–4	Reference	.	.	5.33	88
	5–7	−0.086	0.016	< 0.0001	5.24	126
	8–10	−0.19	0.017	< 0.0001	5.14	149
SCC2022	1–4	Reference	.	.	5.27	80
	5–7	−0.013	0.017	0.45	5.26	107
	8–10	−0.078	0.018	< 0.0001	5.19	176
LOWSCCACHIEVABLE	≤ 3	Reference	.	.	5.33	26
	4	−0.11	0.034	0.0016	5.22	74
	5	−0.16	0.033	< 0.0001	5.17	263
HIGHSCCFROM	Milking machine	Reference	.	.	5.25	6
	Freshly calved cows	0.015	0.045	0.74	5.26	62
	Older cows	−0.013	0.046	0.78	5.23	35
	Housing	−0.036	0.044	0.40	5.21	122
	Grassland	0.027	0.044	0.54	5.27	90
	Unknown source	−0.026	0.048	0.59	5.22	22
	Other	−0.024	0.047	0.61	5.22	26
SCCADVICE	Veterinary professional	Reference	.	.	5.24	68
	Advisory services	0.083	0.026	0.0013	5.32	21
	Peer to peer communication	−0.041	0.026	0.12	5.19	21
	Self-directed learning	0.0078	0.032	0.81	5.24	13
	Veterinary professional and advisory services	−0.0046	0.019	0.82	5.23	49
	Veterinary professional and peer to peer communication	−0.0031	0.021	0.89	5.23	36
	Veterinary professionals and self-directed learning	0.012	0.029	0.68	5.25	16
	Advisory services and peer to peer communication	0.049	0.029	0.091	5.28	16
	Peer to peer communication and self-directed learning	0.040	0.061	0.52	5.27	3
	Veterinary professional, advisory services, and peer to peer communication	−0.041	0.019	0.036	5.19	51
	Veterinary professional, advisory services, and self-directed learning	−0.068	0.033	0.037	5.17	12
	Veterinary professional, peer to peer communication, and self-directed learning	−0.0063	0.026	0.81	5.23	22
	Advisory services, peer to peer communication, and self-directed learning	0.035	0.041	0.40	5.27	7
	Veterinary professional, advisory services, peer to peer communication, and self-directed learning	−0.0050	0.024	0.83	5.23	28

## Discussion

Studies have examined milking management practices and their various relationships with BTSCC on Irish dairy farms [10, 38, 49], and others have documented the prevalence of certain technologies in the absence of associations with BTSCC [50], but none have examined both using data from the same farms. This study contributes to current knowledge by drawing associations between both the manual and technological aspects of milking management and their relative contribution to the BTSCC of the farm which employs them.

### Farm management practices

#### Calving systems

The majority of Irish milk production is based on seasonal calving systems, where farmers calve their cows in spring, in order to maximise the utilisation of grass as a primary food source [51]. Split-calving herds calve their cows in both spring and autumn, in order to maintain a fresh milk supply during the dry-period of seasonally calved cows. In our study, calving pattern showed a significant association with log10BTSCC; with seasonal calving patterns associated with the lowest log10BTSCC. O’Connell et al. [16] reported similar findings, with monthly BTSCC being lower for spring-calving herds than for split-calving herds for the months of February to September and the converse being true for the remaining four months. Milk from herds on a split-calving pattern demonstrated a higher average BTSCC in early and mid-lactation, when the cows in the winter-calving cohort are in late lactation, due to the mixing of milk from different stages of lactation [16]. Differences in management could also contribute to differences in BTSCC, with a study by Horan et al. [52] demonstrating differences in group management and nutritional composition of diets during the transition period for both systems; a period critical to the success of the following lactation [53, 54].

#### Mastitis treatment records

The keeping of mastitis treatment records was also associated with significantly lower log10BTSCC. The use of on-farm records allows vets and farmers alike to make informed decisions about individual animal care and to indicate patterns in mastitis source and spread [55]. It is also a crucial component in the ability of veterinarians to make an informed decision around SDCT [56]. Being able to effectively identify cows affected by chronic high cell count or clinical mastitis contributes to decreased BTSCC through targeted management of these animals within the milking herd; reducing the likelihood of new IMI occurrence and allowing for more informed and successful dry cow therapy decision-making [57].

#### Duration of milking

Our study found that increased duration of morning milking was associated with an increase in log10BTSCC, though the effect size was particularly low. Our findings may reflect the length of time cows are waiting to be milked; increasing risk of stress whilst navigating to the parlour [58] and risk of contamination of teat skin with urine and faeces accumulated in the collecting yard [10, 59]. This supports the findings of Schreiner and Ruegg [60] and Reneau et al. [61] which reported a definitive link between increased rate of subclinical mastitis and increasingly poor cow cleanliness, with Shreiner and Ruegg [60] proposing that the primary sources of exposure for environmental mastitis pathogens were the presence of moisture, mud or manure on leg and udder skin. These findings could also relate to larger herds having longer milking durations, and larger herds (> 73 cows) had a higher monthly average BTSCC than smaller herds (< 73 cows) in our original survey study [44]. However, a study by Prendergast et al. [62] found that for herringbone parlours, a 63% increase in herd size (from 125 to 204 cows) increased total milking process time by only 12%. Larger dairy operations generally had more cows milked per operator per hour, indicating less time spent per cow [62], which is another factor that could contribute to increased BTSCC on larger farms.

#### Individual who conducts milking

The person conducting the milking was also found to be significantly associated with log10BTSCC, with our findings showing that employees milking alone were associated with the highest log10BTSCC. Schewe et al. [41] suggested that employee management may impede mastitis control in larger herds, with their findings showing that farms which ensured strict protocol compliance and enforced penalties to employees in the event of BTSCC increases were more likely to have lower BTSCC. Regarding the importance of family involved in the dairy operation, a study by Contzen and Häberli [63] found that family and stable relationships were “most important” in terms of farmer quality of life; a sentiment that has always been at the heart of Irish dairy [64].

### Parlour management and parlour technologies

#### Parlour type

Our study found that rotary parlours were associated with a higher log10BTSCC than either swing-over herringbones, parallel parlours or herringbones with recording jars. Though there is nothing in literature to support this, we can speculate that rotary parlours do not offer the milker the same ability to visually inspect the condition of the teats particularly post-milking compared to conventional parlours, thus hindering the ability of the milker to identify early signs of mastitis [65] which in turn can lead to increases in BTSCC. Rotary parlours also had more technological add-ons than any of the other types of parlour [44], which may further exacerbate a ‘hands-off’ approach to milking and therefore missed opportunities to identify problem cows. A study by Prendergast et al. [66] identified that rotary farms milked more cows per operator per hour than herringbones parlours and did not practice pre-milking routines.

#### Cow positioning

The presence of straight breast rails in parlours showed an association with increased log10BTSCC. Straight breast rails are added to parlours to assist with cow positioning. Poor alignment of the milking unit respective to the teats, i.e., the cluster not sitting squarely underneath the udder, contributes to unequal quarter emptying, which in turn increases the risk of over-milking of individual quarters [67]. Higher vacuums during over-milking also cause tissue changes around the teat canal, and/or teat-end hyperkeratosis, which can facilitate the harbouring and entry of bacteria into the mammary gland and, consequently, mastitis [68]. This could be a contributory factor to the association of the presence of straight breast rails with increased log10BTSCC in our study, though it is debated in literature as to the degree to which hyperkeratosis affects SCC [69]. Evidence suggests that mostly severe teat-end hyperkeratosis (defined as a ‘3’ or ‘4’ on the scoring criteria of Mein et al. [70]) is a risk factor for subclinical mastitis. Severe teat-end hyperkeratosis is also a strong risk factor for *Staphylococcus aureus* infection; one of the major and most virulent pathogens implicated in subclinical mastitis [71] and the most prevalent pathogen isolated from quarters of cows in Irish dairy herds investigated by McParland et al. [72] and Clabby et al. [38].

#### Cow comfort in the milking parlour

Discomfort or nervousness in the general milking environment has been linked with reduced oxytocin levels, thus inhibiting milk let-down and increasing the risk of vacuum-induced teat tissue changes as previously described [73]. Stress in the milking environment could also be a contributory factor towards the association between the presence of backing gates in the collecting yard and increased log10BTSCC. The purpose of a backing gate is to encourage cows to move into the milking parlour by reducing available space in the collecting yard. Cows walk relatively slowly, with literature stating a non-lame cow walks at a pace of between 0.84 m/s [74] and 1.42 m/s [75]. If cows are rushed by use of a backing gate, it can increase stress and therefore adversely affect the levels of oxytocin required for proper milk let-down. This can lead to bimodal milk flow profiles, longer milking times and increased rates of over-milking with subsequent teat damage and increased SCC through similar methods as described previously for straight breast rails [30, 67, 76].

#### Parlour technology add-ons

Both the presence of ACRs and the presence of automatic washers on the milking machine were associated with decreased log10BTSCC. ACRs are a technology used in milking parlours to automate the process of removing the milking clusters from cows at the end of milking by automatically removing the milking cluster from the cow’s udder once milk flow has decreased below a certain pre-set threshold value (generally 0.2 kg/min), known as the ‘milk flow switch point’. This not only saves time for the farmer, but also reduces stress on the cow and can help prevent over-milking [77]. A study by Upton et al. [78] found that increasing the milk flow switch point (from 0.2 kg/min to 0.8 kg/min at the udder level) showed significantly less leg movement during longer morning milking times. Further studies showed that increasing cluster remover threshold settings from 0.2 kg/min to 0.4 kg/min decreased milking duration with no effect on production, incidence of clinical mastitis, or adverse effect on teat condition, regardless of whether cows were milked with a comprehensive pre-milking routine or not [79, 80]. Though there is little to support the association of automatic washers on the milking machine and decreased log10BTSCC specifically, there is plenty of evidence to highlight the importance of good milking machine sanitation on total bacterial counts (TBC) of raw milk. A positive correlation was observed in a study by Berry et al. [81] between SCC and TBC, indicating that a reduction in BTSCC was generally associated with reduced bulk tank TBC. This likely reflects the association found in our study, in that farmers who have installed an automatic washer on their milking machine are likely more informed about the importance of milking machine hygiene than those without and are able to achieve better consistency of washing with an automated system. These farmers may be more likely to take additional precautions such as pre-milking teat disinfection, which reduces the numbers of bacteria on teat skin prior to teat cup attachment [82], in order to reduce the risk of bacteria being introduced to the milking machine pipeline, thereby reducing both SCC and TBC concurrently; supporting the findings of Berry et al. [81].

#### Cluster disinfection

Conducting cluster disinfection was also associated with lower log10BTSCC, a finding consistent with that of Galton [83] who demonstrated that disinfection of milk liners decreased bacteria counts and incidence of IMI in experimentally challenged cows, albeit this was done using automatic post-milking teat dip via the milking machine. Liners can act as a fomite for contagious mastitis-causing bacteria [84] and, as they are the only element of the milking machine in direct contact with the cow’s teat, preventing accumulation of bacteria within them is advisable to reduce the incidence of new IMI. Automated means of cluster disinfection as a standalone system [85] or with a combined automatic within-cluster post-dipping teat disinfection system [19, 86], assist in achieving this.

### Milking management practices

#### Fore-milking

Discarding of the first few streams of milk from each quarter is considered an effective stimulus for milk let-down [87], and is also beneficial for clinical mastitis detection as it allows farmers to identify any visible abnormalities in the milk, such as blood or clots [24]. We found that fore-milking as part of a milking routine and after calving were both associated with a significant decrease in log10BTSCC compared to never fore-milking at all, which may be expected as not conducting fore-milking may contribute to slower identification of clinical mastitis cases and therefore increased BTSCC. With regard to calving, research shows that the postpartum period in cattle is associated with a physiologically elevated fore-milk SCC [88, 89], with the largest reduction of SCC occurring in the first two weeks after calving [90] and a more rapid decline observed in uninfected quarters [91, 92]. Failure to administer antibiotic therapy at dry-off to appropriate cows [38], too high a milk yield prior to dry-off [93], improper dry cow management in terms of housing [94] and nutrition [95], and physiological failings in ensuring teat closure [96] can further contribute to increased risk of IMI during the dry period and either clinical mastitis incidence or increased SCC in the new lactation. Identifying these cows and prioritising cure as early as possible in the new lactation period will likely reduce BTSCC [96]. With regard to routine fore-milking, a study by Peeler et al. [97] found an increased incidence of clinical mastitis in farms that conducted fore-milking. It is possible that checking fore-milk could result in detection of mild mastitis cases that would otherwise not have been diagnosed, thus increasing the reported incidence, or that fore-milking could increase exposure of other cows to mastitis pathogens in stripped milk, similar to how transmission occurs via leaked milk or through contamination of the cow’s teats from the milkers’ hands. The degree to which stripping foremilk increases transmission of mastitis pathogens in the parlour, rather than increasing detection of mild cases of clinical mastitis, is not currently known. Conducting fore-milking was not directly associated with reduced BTSCC in the literature, but has proven contributions towards a higher two-minute milk yield, a lower low milk flow period, less exhibition of bimodal milk letdown and lower odds of short-term teat tissue changes [98]; all factors which contribute to better teat health.

#### Pre-milking udder preparation

In terms of pre-milking udder preparation, conducting no preparation at all was associated with an increase in log10BTSCC compared to using a combined disinfection and drying step. Pre-milking teat disinfection has been shown to reduce bacterial numbers on teat skin [99, 100] and, regardless of udder cleaning procedure, manual drying of teats has been demonstrated as a significant factor in reduction of total bacteria counts on teat skin [101]. Interestingly, we found that washing udders alone prior to milking was associated with a significant decrease in log10BTSCC compared to a disinfection step alone. This was contrary to the findings of Gleeson et al. [100] which found that while the practice of washing and drying teats reduced bacterial numbers compared to not cleaning teats at all, it could not be considered as effective as cleaning with disinfectant products, especially in the absence of a drying step. Washing is generally discouraged without adequate drying afterwards as pathogens are distributed over the surface of the udder and teats [102]. When washing is required, only teats should be washed, using minimal water, and should be thoroughly dried afterwards [24]. This association is not something to be encouraged in normal milking practice as it cannot be explained rationally, unless it was conducted in a very low challenge environment where the risks of contribution to high BTSCC were minimal.

#### Post-milking teat disinfection

No PMTD was associated with an increased log10BTSCC compared to either spraying or dipping, a finding supported by multiple studies which show that the utilization of an effective PMTD routine during lactation can significantly reduce the incidence of new IMI [102–104]. A comprehensive review of PMTD by Pankey et al. [105] stated that “post-milking teat antisepsis [was] regarded as the single most effective practice for prevention of IMI of lactating dairy cows”. The additional finding of automatic in-cluster dipping being associated with the lowest log10BTSCC may be artificially inflated due to the fact that only two herds practiced it, but it has been proven to be superior to either dipping or spraying in literature when used in combination with a cluster flushing system in terms of reducing BTSCC [19]. In general, the literature supports superiority of dipping compared to spraying in terms of teat coverage [11].

#### Udder hygiene

In terms of udder hygiene maintenance, a combination of clipping tails and clipping udders was associated with the highest log10BTSCC, though it is not entirely clear why this is. It could be speculated that the practice of clipping udders may increase risk of irritation or injury to teat skin; disrupting the first line of defence against mastitis pathogens [106, 107], particularly in unhygienic environments. Milk from cows with udder hygiene scores of ‘3’ or ‘4’ (dirty or very dirty) out of ‘4’ were 150% more likely to contain isolates of major pathogens than cows with scores of ‘1’ and ‘2’ in a study by Schreiner & Ruegg [60]. Barkema et al. [11] found that udder hair was more often clipped every year on low BTSCC farms (≤ 150,000 cells/ml) than medium (150–250,000 cells/ml) or high (250–400,000 cells/ml) BTSCC farms. Dufour et al. [99] also found that frequent clipping or flaming of udder hairs was consistently associated with lower herd SCC. Conversely, the findings of Silk et al. [108] showed that removal of hair surrounding the teat had no effect on the number, nor types, of bacteria that were present on the teat skin of their study herd (mean BTSCC < 150,000 cells/ml). They proposed that it was rather the pre-milking preparation techniques implemented, which included pre-dipping, that effectively removed teat skin surface bacteria, even with hair surrounding the control teats.

### SCC control strategies

#### Milk recording

Our study found that farmers who did not conduct milk recording had a significantly higher log10BTSCC than farmers who conducted milk recording once or eleven times in 2021. Records assist farm managers in assessing target performance progress, in design, monitoring, implementation, and evaluation of management plans, and detection and diagnosis of emerging management and health problems [109]. SCC values from both the last milk recording before drying-off and the first milk recording following calving can be used to illustrate the dynamics of IMI during the dry period [93]. Lactation yields, commonly expressed on a 305-day basis or as a difference from herd mates, and cow ranking based on estimated producing abilities, also provide valid cow comparisons for culling [109]. The prevalence of milk recording in Ireland is lower than some of our European counterparts, with 64% of Irish farmers engaging with it in 2023 (albeit up from 44% in 2021) as opposed to 90% in The Netherlands and 98% in Norway [110]. Milk recording has been associated with low herd-level SCC [10], possibly due to increased farmer knowledge on the health status of individual cows and their subsequent ability to make tailored management decisions on these animals. Balaine et al. [35] concluded that increasing milk recording’s adoption rates would be valuable in increasing output and enhancing animal health for farmers who are not currently doing so.

#### Selective dry cow therapy

Irish farmers are advised to use milk recording results to identify cows that qualify for selective dry cow therapy (SDCT) [111], though we found that farmers often use a combination of factors when making these decisions. SDCT involves only cows or quarters with existing IMI being selected for treatment with intramammary antimicrobials during dry-off, in accordance with the Veterinary Medicines Regulation EU 2019/6 [112] which came into effect in January 2022. We found that respondents who did not utilise any of the listed resources in making SDCT decisions had a higher log10BTSCC than those who used records of clinical mastitis, and combinations of clinical records with milk yield records, individual cow records, individual cow factors (such as age, temperament, etc.) and CMT testing. CMT testing has relatively poor sensitivity in identifying infected cows or infected quarters (approximately 70% and 50%, respectively) [113, 114], but it can be a useful tool at dry-off in herds that are not under major mastitis pathogen pressure [115, 116], and likely have a lower BTSCC. The herds not using any of the listed resources, and therefore possibly not conducting SDCT, may not do so due to the fact that they have a higher BTSCC and/or insufficient records to properly implement selective antibiotic treatment, thus contributing to the association found here.

### Farmer demographics

#### Years in dairy

We found that farmers milking less than ten years had a significantly lower log10BTSCC than those milking more than twenty years. According to literature, young farmers are more likely to be innovative, to farm at a greater intensity, to have larger holdings, and be better educated than older generations [117, 118], which may contribute to lower BSTCC; though the duration of dairy career here does not necessarily reflect farmer age. However, literature shows that increasing age, reflected in the number of years dairying, has a negative effect on adoption rates of technological innovations as older farmers are either unwilling or unable to adapt [119–121], despite proven benefits of many technologies for udder health and BTSCC as described previously.

#### Sources of SCC advice

Good communication between farmers and their veterinary professionals is paramount in influencing compliance with mastitis control programmes [122], and this was reflected in the fact that combinations of where farmers sourced their advice were associated with lower log10BTSCC when a veterinary professional was involved. The well-communicated advice of a veterinary professional would contribute to both the identification of possible udder health threats as well as reassurance of the benefits of proposed interventions that aim to reduce BTSCC. Providing farmers with an opportunity to voice concerns or seek clarifications on issues that are confusing or troubling to them, without necessarily pushing an agenda on them, ensures good compliance and a more beneficial outcome for all involved [123]. Farmers are not a homogeneous group and cannot be convinced by relying only on educational arguments [124, 125], so the importance of veterinary involvement in tangible intervention is imperative. This is of particular importance during dry-off, reflected by a study conducted by Vilar et al. [126] in which 64.4% of surveyed farmers stated they made a decision on DCT based on their own experience, compared to 34.8% on the advice of veterinary professionals and only 0.8% on the advice of peers.

### Study limitations

Though we discovered many significant associations, the effect size was often low, implying that not all of the results may be of practical significance. In addition, in the process of combining categories, the effect of a single condition in a combination (e.g., the ‘drying’ condition in ‘disinfecting and drying’ for pre-milking practices) was impossible to isolate unless that condition was also available for analysis as a sole condition (e.g., ‘drying’ alone for pre-milking practices). This was due to the mutual exclusivity of the single and combination conditions within a given variable. However, though a weakness, this method was deemed the most methodically sound and clinically interpretable way to model the complex, overlapping practices outlined in this manuscript by both university-affiliated veterinary experts and independent statisticians.

## Conclusion

The study examined various farm management practices, parlour management practices and parlour technologies, milking management practices, SCC control strategies and farmer demographic variables to identify their associations with bulk tank somatic cell count (log10BTSCC). Significant associations were found across multiple variables, demonstrating the complexity of factors influencing log10BTSCC. For farm management, utilising seasonal calving patterns, family members milking in conjunction with respondents, and maintaining mastitis treatment records were associated with lower log10BTSCC. Conversely, longer milking durations and certain parlour designs, such as rotary parlours or parlours with additions of straight breast rails or backing gates in their collecting yards, were associated with higher log10BTSCC. Technological advancements such ACRs and automatic washers on the milking machine were associated with significantly reduced log10BTSCC. Effective milking practices, including fore-milking routines and comprehensive pre-milking udder preparation and post-milking teat disinfection, also contributed to lower log10BTSCC. Selective dry cow therapy decisions made using multiple resources was associated with significantly lower log10BTSCC, emphasizing the importance of strategic herd health management; particularly when veterinary involvement was sought with regard to SCC advice. Farmer demographics revealed that level of experience in the dairy industry and confidence levels in their BTSCC control influenced log10BTSCC, with newer farmers and those indicating confidence with regard to achieving a BTSCC of < 200,000 cells/ml showing lower bulk tank cell counts. This comprehensive analysis highlights critical areas for intervention and optimisation in order to improve milk quality and dairy cow welfare through reduced somatic cell counts. There is scope for further research on many of the aspects found to be in association with log10BTSCC in this study, particularly in the areas of cow positioning within parlour design, fore-milking practices, milk recording, and means of disseminating SCC advice to farmers; particularly around the topics of parlour hygiene and selective dry cow therapy.

## Supplementary Information

Supplementary Material 1.

Supplementary Material 2.

## Data Availability

No datasets were generated or analysed during the current study.

## Appendix

**Table:** Monthly average farm bulk milk production data for 12 months of 2021, 8 months of 2022 and combined 20 months (from milk processor dataset)

**Variable Label**	**2021**		**2022**		**Combined**	
	Mean / Median^1^	Std Dev / IQR^2^	Mean / Median^1^	Std Dev / IQR^2^	Mean / Median^1^	Std Dev / IQR^2^
SCC (x1000 cells/ml)	150	103	137	108	145	105
Milk volume (L)	65,082	56,031	75,159	60,745	69,087	58,155
Fat %	4.5	0.5	4.2	0.4	4.4	0.5
Protein %	3.7	0.3	3.5	0.2	3.6	0.3
Total Milk Solids (kg)	5,332	4,561	5,983	4,919	5,591	4,717
Total Dairy Cows	129	92	134	96	131	93

^1^Median values for SCC

^2^Interquartile range values for SCC

**Table:** Spearman’s correlation coefficient scale

Scale of correlation coefficient	Value
0 < ρ ≤ 0.19	Very weak correlation
0.2 ≤ ρ ≤ 0.39	Weak correlation
0.4 ≤ ρ ≤ 0.59	Moderate correlation
0.6 ≤ ρ ≤ 0.79	Strong correlation
0.8 ≤ ρ ≤ 1.0	Very strong correlation

**Table:** Cramer’s V correlation

Cramer’s V value	Value
≥ 0.1	Weak association
≥ 0.3	Moderate association
≥ 0.5	Strong association

**Table:** Pearson’s correlation coefficient scale

Scale of correlation coefficient	Value
0 < r ≤ 0.19	Very low correlation
0.2 ≤ r ≤ 0.39	Low correlation
0.4 ≤ r ≤ 0.59	Moderate correlation
0.6 ≤ r ≤ 0.79	High correlation
0.8 ≤ r ≤ 1.0	Very high correlation

**Table:** Results of the univariate analysis of the significant variables (*p* = ≤0.1) included in the initial farm-specific management multivariable model

Effect		Estimate	Standard Error	*p*-value
CALVINGPATTERN	Seasonal	Reference	.	.
	Split	0.083	0.026	0.0018
	Other	0.11	0.023	<0.0001
MILKER	Respondent conducts milking themselves	Reference	.	.
	Family of respondent conducts milking	0.025	0.036	0.48
	Employee(s) of respondent conducts milking	0.042	0.047	0.36
	Respondent and family of respondent conduct milking	-0.044	0.019	0.021
	Respondent and employee(s) of respondent conduct milking	0.030	0.024	0.21
	Family and employee(s) of respondent conduct milking	0.035	0.064	0.59
	Respondent, family of respondent and employee(s) of respondent conduct milking	-0.0056	0.025	0.82
AGEMILKINGSYSTEM	<5 years	Reference	.	.
	5-10 years	0.066	0.021	0.0023
	11-15 years	0.062	0.024	0.0098
	16-20 years	0.056	0.027	0.039
	21-25 years	0.035	0.029	0.23
	26-30 years	0.075	0.031	0.018
	31-35 years	0.11	0.051	0.039
	36+ years	0.043	0.033	0.19
CULLING	Age	Reference	.	.
	Behaviour	-0.093	0.038	0.015
	Fertility	-0.066	0.024	0.007
	Lameness	-0.035	0.034	0.30
	Poor milk production	-0.015	0.039	0.70
	Recurrent incidences of clinical mastitis	-0.083	0.029	0.005
	Persistently high SCC	-0.047	0.027	0.081
MILKINGFREQUENCY	Once per day	-0.16	0.062	0.011
	Twice per day	-0.19	0.075	0.011
	Voluntary milking / AMS system	-0.19	0.10	0.065
	Other	-0.16	0.062	0.011
AMDURATION		0.0011	0.00025	<0.0001

**Table:** Results of the univariate analysis of the significant variables (*p* = ≤0.1) included in the initial parlour-specific management and parlour technologies multivariable model

Effect		Estimate	Standard Error	*p*-value
PARLOURTYPE	Swing-over herringbone	Reference	.	.
	Automatic milking system (AMS)	-0.043	0.045	0.33
	Double-up herringbone	0.051	0.034	0.13
	Herringbone with recording jars	-0.035	0.018	0.054
	Parallel	-0.035	0.030	0.24
	Rotary	0.062	0.045	0.17
	Other	-0.021	0.053	0.70
STRAIGHTBRRAIL	No straight breast rails	Reference	.	.
	Straight breast rails	0.068	0.028	0.017
MANBAILING	No manual bailing system	Reference	.	.
	Manual bailing system	-0.039	0.021	0.071
AUTOCLUSREM	No automatic cluster removers	Reference	.	.
	Automatic cluster removers	-0.036	0.015	0.015
AUTOWASHMM	No automatic washer on the milking machine	Reference	.	.
	Automatic washer on the milking machine	-0.037	0.015	0.016
BACKINGGATES	No backing gates in the collecting yard	Reference	.	.
	Backing gates in the collecting yard	0.060	0.021	0.0055
VARSPEEDVACC	No variable speed vacuum drive	Reference	.	.
	Variable speed vacuum drive	-0.031	0.016	0.063
NOADDONS	No technological add-ons	Reference	.	.
	At least one technological add-on	0.065	0.034	0.058
LINERCHANGE	Once per year	Reference	.	.
	Twice per year	-0.035	0.016	0.034
	Three times per year	0.068	0.035	0.055
	Every 2 years	-0.075	0.030	0.013
	Every 2000 milkings	0.016	0.063	0.80
	Every 2500 milkings	-0.038	0.031	0.22
	Other	-0.035	0.050	0.49
CLUSTERDISINFECT	Cluster disinfection; no	Reference	.	.
	Cluster disinfection; yes	-0.044	0.015	0.0035

**Table:** Results of the univariate analysis of the significant variables (*p* = ≤0.1) included in the initial milking management multivariable model

Effect		Estimate	Standard Error	*p*-value
FOREMILK	Never fore-milk	Reference	.	.
	Fore-milk as part of milking routine	-0.078	0.035	0.028
	Fore-milk for clinical mastitis indications	-0.036	0.036	0.33
	Fore-milk for subclinical mastitis indications	-0.025	0.051	0.62
	Fore-milk after calving	-0.16	0.056	0.005
	Fore-milk as part of milking routine and for clinical mastitis indications	-0.12	0.076	0.12
	Fore-milk for clinical mastitis indications and subclinical mastitis indications	-0.019	0.039	0.62
	Fore-milk for clinical mastitis indications and after calving	-0.047	0.035	0.18
	Fore-milk for subclinical mastitis indications and after calving	0.099	0.086	0.25
	Fore-milk for clinical mastitis indications, subclinical mastitis indications, and after calving	-0.022	0.035	0.53
	Fore-milk as part of milking routine, for clinical and subclinical mastitis indications, and after calving	-0.098	0.086	0.26
PREMILK	No pre-milking udder preparation	Reference	.	.
	Drying step	-0.020	0.019	0.28
	Wash step	-0.034	0.039	0.38
	Disinfection step	0.018	0.026	0.48
	Mechanical intervention	-0.086	0.057	0.13
	Wash and drying step	-0.003	0.030	0.92
	Disinfection and drying step	-0.083	0.025	0.001
	Wash and disinfection step	0.065	0.064	0.31
	Wash step, disinfection step and drying step	0.031	0.054	0.57
POSTMILK	None	Reference	.	.
	Spraying	-0.022	0.030	0.47
	Dipping	-0.21	0.099	0.032
	Automatic in-cluster dipping	0.13	0.043	0.0020
UDDERHYGIENE	Clip udders	Reference	.	.
	Flame udders	-0.057	0.081	0.48
	Clip tails	0.008	0.053	0.89
	Clip tails and clip udders	0.064	0.056	0.25
	Clip tails and flame udders	-0.012	0.057	0.84
	Clip tails, clip udders and flame udders	-0.029	0.067	0.67

**Table:** Results of the univariate analysis of the significant variables (*p* = ≤0.1) included in the initial SCC control multivariable model

Effect		Estimate	Standard Error	p-value
RECORDSKEPT	Whiteboard	Reference	.	.
	Farm recording book	-0.049	0.027	0.065
	App	-0.065	0.026	0.013
	Whiteboard and farm recording book	-0.065	0.029	0.027
	Whiteboard and app	-0.099	0.029	0.0010
	Farm recording book and app	-0.090	0.043	0.039
	Whiteboard, farm recording book and app	-0.091	0.045	0.046
TSONLY	None of the listed resources	Reference	.	.
	Records of clinical cases throughout lactation	-0.080	0.035	0.022
	Milk yield records	-0.090	0.041	0.030
	Cow factors	0.072	0.053	0.17
	CMT testing	0.0010	0.064	0.99
	Individual cow records	-0.060	0.029	0.044
	Records of clinical cases and milk yield records	-0.11	0.048	0.025
	Records of clinical cases and cow factors	-0.19	0.070	0.0060
	Records of clinical cases and individual cow records	-0.11	0.031	0.0004
	Milk yield records and cow factors	0.022	0.070	0.76
	Milk yield records and individual cow records	-0.036	0.098	0.71
	Cow factors and individual cow records	-0.048	0.070	0.50
	Records of clinical cases, milk yield records, and cow factors	-0.11	0.060	0.066
	Records of clinical mastitis, milk yield records, and individual cow records	-0.0010	0.051	0.99
	Records of clinical mastitis, cow factors, and individual cow records	-0.12	0.035	0.001
	Records of clinical mastitis, CMT testing, and individual cow records	-0.11	0.044	0.014
	Cow factors, CMT testing, and individual cow records	0.0030	0.080	0.98
	Records of clinical cases, milk yield records, cow factors, and individual cow records	-0.039	0.050	0.44
	Records of clinical cases, milk yield records, CMT testing, and individual cow records	0.051	0.095	0.60
	Records of clinical cases, cow factors, CMT testing, and individual cow records	-0.098	0.043	0.024
	Records of clinical cases, milk yield records, cow factors, CMT testing, and individual cow records	-0.17	0.046	0.0002
MILKRECORDINGS	0	Reference	.	.
	1	-0.074	0.070	0.30
	2	0.063	0.070	0.37
	3	0.063	0.032	0.050
	4	-0.024	0.020	0.25
	5	-0.046	0.023	0.047
	6	-0.017	0.028	0.56
	7	-0.060	0.030	0.048
	8	-0.11	0.070	0.10
	9	-0.21	0.070	0.0030
	10	0.058	0.070	0.41
	11	-0.19	0.069	0.0060

**Table:** Results of the univariate analysis of the significant variables (*p* = ≤0.1) included in the initial farmer demographic multivariable model

Effect		Estimate	Standard Error	p-value
YEARSDAIRYING	<5 years	Reference	.	.
	5-10 years	0.051	0.038	0.18
	10-20 years	0.074	0.037	0.047
	20-30 years	0.071	0.035	0.044
	30-40 years	0.072	0.036	0.046
	40+ years	0.12	0.036	0.0010
SCC2021	1-4	Reference	.	.
	5-7	-0.12	0.016	<0.0001
	8-10	-0.25	0.015	<0.0001
SCC2022	1-4	Reference	.	.
	5-7	-0.096	0.017	<0.0001
	8-10	-0.22	0.016	<0.0001
LOWSCCACHIEVABLE	≤ 3	Reference	.	.
	4	-0.15	0.039	<0.0001
	5	-0.30	0.037	<0.0001
REPORTUSEFULNESS	≤ 3	Reference	.	.
	4	0.011	0.027	0.68
	5	-0.053	0.025	0.034
LEGISLATIONEFFECT	1-4	Reference	.	.
	5-7	0.044	0.018	0.015
	8-10	0.078	0.018	<0.0001
CONFIDENCESDCT	1-4	Reference	.	.
	5-7	-0.049	0.018	0.0060
	8-10	-0.12	0.016	<0.0001
HIGHSCCFROM	Milking machine	Reference	.	.
	Freshly calved cows	0.0050	0.059	0.93
	Older cows	-0.023	0.060	0.70
	Housing	-0.060	0.057	0.30
	Grassland	0.029	0.058	0.61
	Unknown source	0.0080	0.063	0.90
	Other	-0.072	0.062	0.24
SCCADVICE	Veterinary professional	Reference	.	.
	Advisory services	0.10	0.034	0.0030
	Peer to peer communication	-0.054	0.034	0.12
	Self-directed learning	0.047	0.042	0.27
	Veterinary professional and advisory services	0.011	0.026	0.66
	Veterinary professional and peer to peer communication	0.032	0.028	0.25
	Veterinary professionals and self-directed learning	0.035	0.038	0.36
	Advisory services and peer to peer communication	0.017	0.039	0.65
	Peer to peer communication and self-directed learning	0.0010	0.082	0.99
	Veterinary professional, advisory services, and peer to peer communication	-0.010	0.025	0.70
	Veterinary professional, advisory services, and self-directed learning	-0.079	0.043	0.070
	Veterinary professional, peer to peer communication, and self-directed learning	0.014	0.034	0.68
	Advisory services, peer to peer communication, and self-directed learning	-0.011	0.055	0.84
	Veterinary professional, advisory services, peer to peer communication, and self-directed learning	0.020	0.031	0.51

## Abbreviations

BTSCC: Bulk tank somatic cell count
SCC: Somatic cell count
log10BTSCC: Log-10 transformed bulk tank somatic cell count
TASAH: Targeted advisory service on animal health
ACR: Automatic cluster remover
PMTD: Post-milking teat disinfection
IMI: Intramammary infection
DCT: Dry cow therapy
ITS: Internal teat sealant
ICBF: Irish Cattle Breeding Federation
CMT: California mastitis test
AMS: Automatic milking system
SDCT: Selective dry cow therapy
TBC: Total bacterial count
