# Supplementary material for: Associations between on-farm factors and bulk tank SCC on Irish dairy farms
Source: Ir Vet J. 2025 Jul 7;78:15. doi: 10.1186/s13620-025-00300-8 (PMC12235848; doi:10.1186/s13620-025-00300-8)
Supplement: Supplementary file 1 — Supplementary Material 1. [file 13620_2025_300_MOESM1_ESM.docx]

# **Supplementary Materials:**

### **Supplementary materials 1a: AM farm-specific management variables included in initial model (i.e. p = ≤0.1 in univariate analysis) (n=7)**

| **Question** | **Model identifier** | **Variable Type** | **Answers** |
| --- | --- | --- | --- |
| Which calving system do you operate on your farm? | CALVINGPATTERN* | Nominal categorical | Seasonal  Split  Other |
| Who are the ‘milkers’ on the farm? Please select one or all that apply to your farm | MILKER* | Nominal categorical | Myself  Family  Employee  (Combinations of any/all of the above) |
| Frequency of milking | MILKINGFREQUENCY | Nominal categorical | Once per day  Twice per day  Voluntary milking / AMS system  Other |
| Normal **morning** average duration (Duration = Time from milking machine turned on until cleaning is complete) | AMDURATION* | Continuous | Average duration of morning milking (minutes) |
| Age of milking system since installation on your farm (**years**); i.e. when the milking system was purchased – not age since last upgrade | AGEMILKINGSYSTEM | Ordinal categorical | <5  5-10  11-15  16-20  21-25  26-30  31-35  36+ |
| For **your** farm, what is the **primary** factor for your decision to cull cows (i.e. score of ‘1’ on a ‘1-7’ scale): | CULLING | Nominal categorical | Age  Behaviour  Fertility  Lameness  Poor milk production  Recurrent incidences of clinical mastitis  Persistently high SCC |
| Do you keep mastitis treatment records? | MASTITISTXRECORDS* | Nominal categorical | Yes  No |

* variable remained in the final model

### **Supplementary materials 1b: AM and PM farm-specific management variables excluded from initial model (i.e. p = >0.1 in univariate analysis) (n=6)**

| **Question** | **Model identifier** | **Variable Type** | **Answers** |
| --- | --- | --- | --- |
| Herd size quartiles | QUARTILES^1^ | Ordinal categorical | Q1 (<73 cows)  Q2 (73-105 cows)  Q3 (106-159 cows)  Q4 (<159 cows) |
| Do you milk in more than one parlour over the course of lactation? | MORETHANONEPARLOUR | Nominal categorical | Yes  No |
| Is your farm managed as part of a partnership? | PARTNERSHIP | Nominal categorical | Yes  No |
| Milking interval; i.e. time between morning milking start time and evening milking start time | MILKINGINTERVAL | Continuous | Hours and minutes (discrete categories) |
| How many people are in the parlour for **morning** milking during peak lactation? If people come and go during milking, count these as a 0.5; e.g. 2.5 | PEOPLEAM | Nominal categorical | Number (discrete categories) |
| How many people are in the parlour for **evening** milking during peak lactation? If people come and go during milking, count these as a 0.5; e.g. 2.5 | PEOPLEPM | Nominal categorical | Number (discrete categories) |

^1^ Excluded from initial model due to observed relationship with log10milklitres

### **Supplementary materials 2a: Parlour-specific management and parlour technology variables included in initial model (i.e. p = ≤0.1 in univariate analysis) (n=10)**

| **Question** | **Model identifier** | **Variable Type** | **Answers** |
| --- | --- | --- | --- |
| Parlour type; i.e. the parlour that accounts for the majority of milkings per year | PARLOURTYPE* | Nominal categorical | Swing-over herringbone  Double-up herringbone  Herringbone with recording jars  Parallel / Side-by-side  Rotary  Robot / Automated Milking System (AMS)  Other (please specify) |
| Which, if any, of the following do you employ to assist with cow positioning in the parlour? | MANBAILING | Nominal categorical | Manual bailing system   - Yes - No |
|  | STRAIGHTBRRAIL* |  | Straight breast rail   - Yes - No |
| Parlour add-ons: Please select from the following list which parlour add-ons you have on your farm | NOADDONS | Nominal categorical | No technological add-ons   - Yes - No |
|  | AUTOCLUSREM* |  | Automatic cluster removers (ACRs)   - Yes - No |
|  | AUTOWASHMM* |  | Automatic washer on the milking machine   - Yes - No |
|  | BACKINGGATES* |  | Backing gates in the collecting yard   - Yes - No |
|  | VARSPEEDVACC |  | Variable speed vacuum pump   - Yes - No |
| Frequency of liner changes | LINERCHANGE | Nominal categorical | Once per year  Twice per year  Three times per year  Every 2 years  Every 2000 milkings  Every 2500 milkings  Other (please specify) |
| Do you employ cluster disinfection on your farm? | CLUSTERDISINFECT* | Nominal categorical | Yes  No |

* variable remained in the final model

### **Supplementary materials 2b: Parlour-specific management and parlour technology variables excluded from initial model (i.e. p = >0.1 in univariate analysis) (n=27)**

| **Question** | **Model identifier** | **Variable Type** | **Answers** |
| --- | --- | --- | --- |
| Parlour manufacturer; Please answer for the parlour that accounts for the majority of milkings each year | PARLOURMANUFACTURER^1^ | Nominal categorical | ATL Agricultural Technology Ltd  BouMatic  Dairymaster  DeLaval  ElectroMech Agri  Fullwood Packo  Gascoigne  G.E.A.  Lely  Milfos  Pearson  Waikato  Hybrid (components from more than one manufacturer)  Other |
| Have you implemented any major upgrades to your milking system in the last **5** years?  E.g. bailing system, ACRs, etc.. | UPGRADES | Nominal categorical | Yes  No |
| How many times **per year** is the milking machine serviced? | SERVICED | Ordinal categorical | Less than once per year  Once per year  Twice per year  More than twice per year |
| Milk line: Where is your milk line situated in the parlour? | MILKLINE | Nominal categorical | Low level (below where the cows are standing)  Mid level (at or above where the cows are standing)  AMS / Robot parlour |
| Pulsation type: Please select from the following options which pulsation type you have on your farm | PULSATION^1^ | Nominal categorical | Simultaneous (4x0): Pressure is applied to, and released from, all teats at the same time  Alternating (2x2): Pressure is applied to two teats while the other two rest and then switches over  One pulsator per teat: Pressure is applied to, and released from, one teat at a time |
| Which, if any, of the following do you employ to assist with cow positioning in the parlour? | SEQBAILING | Nominal categorical | Sequential bailing system   - Yes - No |
|  | INDMANGERS |  | Individual mangers   - Yes - No |
|  | ZIGRURAIL |  | Zig-zag rump rail   - Yes - No |
|  | STRRURAIL |  | Straight rump rail   - Yes - No |
|  | ADBRRAIL |  | Adjustable breast rail   - Yes - No |
| Parlour add-ons: Please select from the following list which parlour add-ons you have on your farm | DUALVACC | Nominal categorical | Dual vacuum systems   - Yes - No |
|  | ACF |  | Automatic cluster flush system (ACF)   - Yes - No |
|  | AUTOCLUDIP |  | Automatic cluster dipping   - Yes - No |
|  | AUTOWASHBT |  | Automatic washer on the bulk tank   - Yes - No |
|  | INPARLOURFEED |  | In-parlour feeding   - Yes - No |
|  | AUTOID |  | Automatic ID detection system   - Yes - No |
|  | AUTOTEATSPRAY |  | Automatic teat sprayers   - Yes - No |
|  | ELECMETERS |  | Electronic milk meters   - Yes - No |
|  | NONELECMETERS |  | Non-electronic milk meters   - Yes - No |
|  | DUMPLINE |  | Milk dump / diversion line   - Yes - No |
|  | AUTOMASTITISDET |  | Automatic mastitis detection system   - Yes - No |
|  | AUTODRAFT |  | Automatic drafting gates   - Yes - No |
|  | VARSPEEDMILKP |  | Variable speed milk pump   - Yes - No |
|  | GATESPIT |  | Entrance / exit gates that can be controlled from the pit   - Yes - No |
| If cluster disinfection is carried out, is it done manually or automatically? | CLUDISAUTOMANUAL | Nominal categorical | Manual  Automatic |
| If manual cluster disinfection, when are clusters disinfected? | MANWHEN | Nominal categorical | After every cow that has been milked  After every high cell count cow that has been milked  Other (please specify) |
| Product(s) used for cluster disinfection – concentrate or pre-mixed? | CONORMIX | Nominal categorical | Concentrate / ‘Make it yourself’  Pre-mixed / ‘Ready to go’ |

^1^ Parlour manufacturer and pulsation were removed from the model due to multiple implausibilities in survey responses despite having p values of <0.1

### **Supplementary materials 3a: Milking management variables included in initial model (i.e. p = ≤0.1 in univariate analysis) (n=4)**

| **Question** | **Model identifier** | **Variable Type** | **Answers** |
| --- | --- | --- | --- |
| When, if ever, do you employ strip milking / fore-milking on your farm?:  Please select all that apply to your farm | FOREMILK* | Nominal categorical | Never  As part of a milking routine (i.e. after every milking, after every morning milking)  For clinical mastitis indications only (i.e. clinically abnormal udder, clots identified in the milk filter)  For subclinical mastitis indications only (i.e. an increase in BTSCC)  After calving  (Combinations of any/all of the above) |
| Pre-milking udder preparation: Please select from the following practices that reflect the **pre-milking** stage of the milking process on your farm | PREMILK* | Nominal categorical | No pre-milking udder preparation  Drying stage (i.e. dry wipe, use of individual or communal cloths used to dry after a washing or disinfecting step)  Washing stage (i.e. use of a hose, washing using an individual or communal udder cloth)  Disinfecting stage (i.e. pre-spraying or pre-dipping)  Mechanical intervention (i.e. pre-milking wash cup or udder brush)  (Combinations of any/all of the above) |
| Post-milking teat disinfection: Please select from the following the practices that reflect the **post-milking** stage of the milking process on your farm | POSTMILK* | Nominal categorical | None  Spraying  Dipping  Automatic in-cluster dipping |
| Udder hygiene: Please select the options that apply to the udder hygiene practices on your farm | UDDERHYGIENE* | Nominal categorical | Clip tails  Clip udders  Flame udders  (Combinations of any/all of the above) |

### **Supplementary materials 3b: Milking management variables excluded from initial model (i.e. p = >0.1 in univariate analysis) (n=4)**

| **Question** | **Model identifier** | **Variable Type** | **Answers** |
| --- | --- | --- | --- |
| If fore-milking is done, it is __? | WHEREFOREMILK | Nominal categorical | Onto the floor  Into a strip cup  Into an ungloved / bare hand  Into a gloved hand |
| Do you conduct CMT testing on your farm to identify high SCC cows? | CMT | Nominal categorical | Yes  No |
| Do you wear gloves in the parlour while milking? | GLOVES | Nominal categorical | Always  Sometimes  Never |
| Please choose the option that best describes your glove-wearing practices in the parlour during milking | GLOVETYPE | Nominal categorical | Disposable rubber gloves  Reusable washable gloves  Bare hands |

### **Supplementary Materials 4a: SCC control variables included in initial model (i.e. p = ≤0.1 in univariate analysis) (n=3)**

| **Question** | **Model identifier** | **Variable Type** | **Answers** |
| --- | --- | --- | --- |
| If you keep mastitis treatment records, how are they kept? | RECORDSKEPT | Nominal categorical | Whiteboard  Farm recording book  App  (Combinations of any/all of the above) |
| If you are using **teat sealant alone** at drying-off in some of your cows, what information do you use to decide this treatment? | TSONLY* | Nominal categorical | None of the listed resources  Records of clinical cases and their outcomes throughout the lactation  Milk yield records  Cow factors (age, teat condition, temperament)  CMT testing  Individual cow records  (Combinations of any/all of the above) |
| How many milk recordings were conducted in 2021? | MILKRECORDINGS* | Ordinal categorical | 0  1  2  3  4  5  6  7  8  9  10  11 |

* variable included in the final model

### **Supplementary Materials 4b: SCC control variables excluded from the initial model (i.e. p = >0.1 in univariate analysis) (n=2)**

| **Question** | **Model identifier** | **Variable Type** | **Answers** |
| --- | --- | --- | --- |
| I milk my high SCC cows.. | MILKHIGHSCC | Ordinal categorical | With the rest of the herd  Before the rest of the herd  After the rest of the herd |
| How often do you engage with bacteriology / culture and sensitivity of milk samples? | BACTERIOLOGY | Nominal categorical | For clinical mastitis only (cows with hot, swollen, painful udders with visible milk abnormalities)  For subclinical mastitis only (cows with a high SCC but normal udder and milk)  For both clinical and subclinical mastitis cows  Only when I am advised by the vet  Rarely  Never |

### **Supplementary Materials 5a: Farmer demographic variables included in initial model (i.e. p = ≤0.1 in univariate analysis) (n=9)**

| **Question** | **Model identifier** | **Variable Type** | **Answers** |
| --- | --- | --- | --- |
| How many years have you spent dairying? | YEARSDAIRYING* | Ordinal categorical | <5  5-10  10-20  20-30  30-40  40+ |
| How did you feel about your overall SCC for **2021**?;  1=”very worried”, 10=”very happy” | SCC2021* | Ordinal categorical | 1-4  5-7  8-10 |
| How do you feel about your overall SCC for **2022** thus far?;  1=”very worried”, 10=”very happy” | SCC2022* | Ordinal categorical | 1-4  5-7  8-10 |
| I believe that a low SCC (<200,000 cells/ml) is achievable on my farm..;  1=”Strongly disagree”, 5=”Strongly agree” | LOWSCCACHIEVABLE* | Ordinal categorical | ≤ 3  4  5 |
| I find that milk recording reports / texts are useful for reducing SCC..;  1=”Strongly disagree”, 5=”Strongly agree” | REPORTUSEFULNESS | Ordinal categorical | ≤ 3  4  5 |
| I seek advice about cell count / SCC from.. | SCCADVICE* | Nominal categorical | Veterinary professional  Advisory services (i.e. co-op milk quality advisor, other advisor or on-site visit by specialist mastitis management experts)  Peer to peer communication (i.e. discussion with colleagues / other farmers)  Self-directed learning (i.e. websites, magazines, handbooks)  (Combinations of any/all of the above) |
| I believe that a high SCC on farms comes **predominantly** from…: Please select one option that you feel is the most related to high SCC on farms in general | HIGHSCCFROM* | Nominal categorical | The milking machine  The milking process / milking practices  Freshly calved cows  Older cows  Housing  Grassland  Unknown source |
| How will the new legislation on antibiotic use affect your current antibiotic usage at dry-off?;  1=”it will not affect it at all”, 10=”it will drastically affect it” | LEGISLATIONEFFECT | Ordinal categorical | 1-4  5-7  8-10 |
| I am confident that I can manage my SCC with selective dry cow therapy..;  1=”not confident at all”, 5=”neutral”, 10=”very confident” | CONFIDENCESDCT | Ordinal categorical | 1-4  5-7  8-10 |

* variable included in the final model

### **Supplementary Materials 5b: Farmer demographic variables excluded from the initial model (i.e. p = >0.1 in univariate analysis) (n=5)**

| **Question** | **Model identifier** | **Variable Type** | **Answers** |
| --- | --- | --- | --- |
| Gender | GENDER | Nominal categorical | Male  Female  Other |
| Age | AGE | Ordinal categorical | 18-24  25-34  35-44  45-54  55-64  65+ |
| Education | EDUCATION^1^ | Nominal categorical | Primary school  Junior Certificate  Leaving Certificate  Certificate in Agriculture (e.g. Green Cert)  Agricultural college: One year  Agricultural college: More than one year  Third-level education: Undergraduate degree  Third-level education: Postgraduate degree  Prefer not to answer |
| Have you availed of a free TASAH Dry Cow Consult? | TASAHDCC^2^ | Nominal categorical | Yes  No |
| On a scale of 1 to 10, I rate my love of being a dairy farmer at a…;  1 to 10 = low to high | CAREERSATISFACTION | Ordinal categorical | 1-4  5-7  8-10 |

^1^ Education was removed from the model due to the sensitive nature of the question and its lack of demonstrated association with log10BTSCC

^2^ TASAH dry cow consultation variable was removed as this service is only available to farmers who have a twelve-month average BTSCC of <200,000 cells/ml and have also conducted a minimum of four whole herd milk recordings over the same timeframe
