# Supplementary material for: Associations between on-farm factors and bulk tank SCC on Irish dairy farms
Source: Ir Vet J. 2025 Jul 7;78:15. doi: 10.1186/s13620-025-00300-8 (PMC12235848; doi:10.1186/s13620-025-00300-8)
Supplement: Supplementary file 2 — Supplementary Material 2. [file 13620_2025_300_MOESM2_ESM.docx]

# **Supplementary Tables:**

### **Supplementary Table 1: Significant associations within farm management variables (difference of least square means moving from category 1 to category 2)**

| **Variable** | **Category 1** | **Category 2** | **Direction of association** | **p-value** |
| --- | --- | --- | --- | --- |
| CALVINGPATTERN | Seasonal | Split | ↑ | 0.0061 |
|  | Seasonal | Other | ↑ | 0.0001 |
| MILKER | Respondent and family conduct milking | Family conducts milking | ↑ | 0.042 |
|  | Respondent and family conduct milking | Employee conducts milking | ↑ | 0.029 |
|  | Respondent and family conduct milking | Respondent and employee conduct milking | ↑ | 0.011 |
|  | Respondent and family conduct milking | Respondent conducts milking | ↑ | 0.027 |

### **Supplementary Table 2: Significant associations within parlour management and technology variables (difference of least square means moving from category 1 to category 2)**

| **Variable** | **Category 1** | **Category 2** | **Direction of association** | **p-value** |
| --- | --- | --- | --- | --- |
| PARLOURTYPE | Herringbone with recording jars | AMS | ↑ | 0.021 |
|  | Herringbone with recording jars | Double-up herringbone | ↑ | 0.0011 |
|  | Herringbone with recording jars | Rotary | ↑ | 0.0006 |
|  | Herringbone with recording jars | Swing-over herringbone | ↑ | 0.0028 |
|  | Parallel | Double-up herringbone | ↑ | 0.039 |
|  | Rotary | Parallel | ↓ | 0.0075 |
|  |  |  |  |  |
|  | Rotary | Swing-over herringbone | ↓ | 0.011 |

### **Supplementary Table 3: Significant associations within milking management variables (difference of least square means moving from category 1 to category 2)**

| **Variable** | **Category 1** | **Category 2** | **Direction of association** | **p-value** |
| --- | --- | --- | --- | --- |
| FOREMILK | Fore-milk as part of a milking routine | Never conduct fore-milking | ↑ | 0.027 |
|  | Fore-milk as part of a milking routine | Fore-milk for clinical mastitis indications and subclinical mastitis indications | ↑ | 0.018 |
|  | Fore-milk as part of a milking routine | Fore-milk for subclinical mastitis indications and after calving | ↑ | 0.048 |
|  | Fore-milk as part of a milking routine | Fore-milk for clinical mastitis indications, subclinical mastitis indications and after calving | ↑ | 0.0020 |
|  | Fore-milk after calving | Never conduct fore-milking | ↑ | 0.031 |
|  | Fore-milk after calving | Fore-milk for clinical mastitis indications and subclinical mastitis indications | ↑ | 0.035 |
|  | Fore-milk after calving | Fore-milk for subclinical mastitis indications and after calving | ↑ | 0.025 |
|  | Fore-milk after calving | Fore-milk for clinical mastitis indications, subclinical mastitis indications and after calving | ↑ | 0.020 |
| PREMILK | Disinfection and drying step | No pre-milking udder preparation | ↑ | 0.0004 |
|  | Disinfection and drying step | Drying step | ↑ | 0.0017 |
|  | Disinfection and drying step | Disinfection step | ↑ | <0.0001 |
|  | Disinfection and drying step | Wash and drying step | ↑ | 0.011 |
|  | Disinfection and drying step | Wash and disinfection step | ↑ | 0.0056 |
|  | Wash step | Disinfection step | ↑ | 0.017 |
|  | Wash step | Wash and disinfection step | ↑ | 0.030 |
| POSTMILK | No post-milking teat disinfection | Spraying | ↓ | 0.034 |
|  | No post-milking teat disinfection | Automatic in-cluster dipping | ↓ | 0.0072 |
|  | Automatic in-cluster dipping | Spraying | ↑ | 0.043 |
|  | Automatic in-cluster dipping | Dipping | ↑ | 0.035 |
| UDDERHYGIENE | Clip tails and clip udders | Flame udders | ↓ | 0.023 |
|  | Clip tails and clip udders | Clip tails | ↓ | 0.0002 |
|  | Clip tails and clip udders | Clip tails and flame udders | ↓ | 0.0021 |
|  | Clip tails and clip udders | Clip tails, clip udders and flame udders | ↓ | 0.0080 |

### **Supplementary Table 4: Significant associations within SCC control variables (difference of least square means moving from category 1 to category 2)**

| **Variable** | **Category 1** | **Category 2** | **Direction of association** | **p-value** |
| --- | --- | --- | --- | --- |
| TSONLY | Records of clinical mastitis throughout lactation | Cow factors | ↑ | 0.0050 |
|  | Records of clinical mastitis throughout lactation | Records of clinical mastitis, milk yield records, cow factors, CMT testing, and individual cow records | ↓ | 0.034 |
|  | Records of clinical mastitis throughout lactation | None of the listed resources | ↑ | 0.026 |
|  | Milk yield records | Cow factors | ↑ | 0.012 |
|  | Milk yield records | Records of clinical mastitis, milk yield records, cow factors, CMT testing, and individual cow records | ↓ | 0.049 |
|  | Cow factors | Individual cow records | ↓ | 0.013 |
|  | Cow factors | Records of clinical mastitis and milk yield records | ↓ | 0.0017 |
|  | Cow factors | Records of clinical mastitis and cow factors | ↓ | 0.001 |
|  | Cow factors | Records of clinical mastitis and individual cow records | ↓ | 0.0009 |
|  | Cow factors | Records of clinical mastitis, milk yield records, and cow factors | ↓ | 0.0019 |
|  | Cow factors | Records of clinical mastitis, cow factors, and individual cow records | ↓ | 0.0007 |
|  | Cow factors | Records of clinical mastitis, CMT testing, and individual cow records | ↓ | 0.0022 |
|  | Cow factors | Records of clinical cases, cow factors, CMT testing, and individual cow records | ↓ | 0.0062 |
|  | Cow factors | Records of clinical cases, milk yield records, cow factors, CMT testing, and individual cow records | ↓ | <0.0001 |
|  | CMT testing | Records of clinical cases, milk yield records, cow factors, CMT testing, and individual cow records | ↓ | 0.031 |
|  | Individual cow records | Records of clinical mastitis, cow factors, and individual cow records | ↓ | 0.038 |
|  | Individual cow records | Records of clinical cases, milk yield records, cow factors, CMT testing, and individual cow records | ↓ | 0.0040 |
|  | Records of clinical cases and milk yield records | Milk yield records and cow factors | ↑ | 0.034 |
|  | Records of clinical cases and milk yield records | Records of clinical mastitis, milk yield records, and individual cow records | ↑ | 0.020 |
|  | Records of clinical cases and milk yield records | None of the listed resources | ↑ | 0.0079 |
|  | Records of clinical cases and cow factors | Milk yield records and cow factors | ↑ | 0.017 |
|  | Records of clinical cases and cow factors | Records of clinical mastitis, milk yield records, and individual cow records | ↑ | 0.014 |
|  | Records of clinical cases and cow factors | Cow factors, CMT testing, and individual cow records | ↑ | 0.045 |
|  | Records of clinical cases and cow factors | Records of clinical cases, milk yield records, CMT testing, and individual cow records | ↑ | 0.026 |
|  | Records of clinical cases and cow factors | None of the listed resources | ↑ | 0.0074 |
|  | Records of clinical cases and individual cow records | Milk yield records and cow factors | ↑ | 0.044 |
|  | Records of clinical cases and individual cow records | Records of clinical mastitis, milk yield records, and individual cow records | ↑ | 0.020 |
|  | Records of clinical cases and individual cow records | None of the listed resources | ↑ | 0.0015 |
|  | Milk yield records and cow factors | Records of clinical cases, milk yield records, and cow factors | ↓ | 0.029 |
|  | Milk yield records and cow factors | Records of clinical mastitis, cow factors, and individual cow records | ↓ | 0.032 |
|  | Milk yield records and cow factors | Records of clinical mastitis, CMT testing, and individual cow records | ↓ | 0.047 |
|  | Milk yield records and cow factors | Records of clinical cases, milk yield records, cow factors, CMT testing, and individual cow records | ↓ | 0.0070 |
|  | Records of clinical mastitis, milk yield records, and cow factors | Records of clinical mastitis, milk yield records, and individual cow records | ↑ | 0.022 |
|  | Records of clinical mastitis, milk yield records, and cow factors | Records of clinical cases, milk yield records, CMT testing, and individual cow records | ↑ | 0.042 |
|  | Records of clinical mastitis, milk yield records, and cow factors | None of the listed resources | ↑ | 0.011 |
|  | Records of clinical mastitis, milk yield records, and individual cow records | Records of clinical mastitis, cow factors, and individual cow records | ↓ | 0.012 |
|  | Records of clinical mastitis, milk yield records, and individual cow records | Records of clinical mastitis, CMT testing, and individual cow records | ↓ | 0.029 |
|  | Records of clinical mastitis, milk yield records, and individual cow records | Records of clinical cases, milk yield records, cow factors, CMT testing, and individual cow records | ↓ | 0.0015 |
|  | Records of clinical mastitis, cow factors, and individual cow records | None of the listed resources | ↑ | 0.0013 |
|  | Records of clinical mastitis, CMT testing, and individual cow records | None of the listed resources | ↑ | 0.010 |
|  | Cow factors, CMT testing, and individual cow records | Records of clinical cases, milk yield records, cow factors, CMT testing, and individual cow records | ↓ | 0.029 |
|  | Records of clinical cases, milk yield records, cow factors, and individual cow records | Records of clinical cases, milk yield records, cow factors, CMT testing, and individual cow records | ↓ | 0.031 |
|  | Records of clinical cases, milk yield records, CMT testing, and individual cow records | Records of clinical cases, milk yield records, cow factors, CMT testing, and individual cow records | ↓ | 0.015 |
|  | Records of clinical cases, cow factors, CMT testing, and individual cow records | None of the listed resources | ↑ | 0.035 |
|  | Records of clinical cases, milk yield records, cow factors, CMT testing, and individual cow records | None of the listed resources | ↑ | 0.0002 |
| MILKRECORDINGS | 1 | 2 | ↑ | 0.0022 |
|  | 1 | 3 | ↑ | 0.0002 |
|  | 1 | 4 | ↑ | 0.011 |
|  | 1 | 5 | ↑ | 0.028 |
|  | 1 | 6 | ↑ | 0.0092 |
|  | 1 | 7 | ↑ | 0.023 |
|  | 1 | 10 | ↑ | 0.0057 |
|  | 1 | 0 | ↑ | 0.013 |
|  | 2 | 8 | ↓ | 0.048 |
|  | 2 | 9 | ↓ | 0.033 |
|  | 2 | 11 | ↓ | 0.0065 |
|  | 3 | 4 | ↓ | 0.0004 |
|  | 3 | 5 | ↓ | <0.0001 |
|  | 3 | 6 | ↓ | 0.0064 |
|  | 3 | 7 | ↓ | 0.0010 |
|  | 3 | 8 | ↓ | 0.0085 |
|  | 3 | 9 | ↓ | 0.0057 |
|  | 3 | 11 | ↓ | 0.0002 |
|  | 3 | 0 | ↓ | 0.0008 |
|  | 4 | 11 | ↓ | 0.020 |
|  | 6 | 11 | ↓ | 0.015 |
|  | 7 | 11 | ↓ | 0.043 |
|  | 10 | 11 | ↓ | 0.011 |
|  | 11 | 0 | ↑ | 0.025 |

### **Supplementary Table 5: Significant associations within farmer demographic variables (difference of least square means moving from category 1 to category 2)**

| **Variable** | **Category 1** | **Category 2** | **Direction of association** | **p-value** |
| --- | --- | --- | --- | --- |
| YEARSDAIRYING | 5-10 years | 20-30 years | ↑ | 0.017 |
|  | 5-10 years | 40+ years | ↑ | 0.049 |
|  | 5-10 years | <5 years | ↓ | 0.020 |
|  | 10-20 years | <5 years | ↓ | 0.0004 |
|  | 20-30 years | <5 years | ↓ | <0.0001 |
|  | 30-40 years | <5 years | ↓ | 0.0005 |
|  | 40+ years | <5 years | ↓ | 0.0001 |
| SCC2021 | 1-4 | 5-7 | ↓ | <0.0001 |
|  | 1-4 | 8-10 | ↓ | <0.0001 |
|  | 5-7 | 8-10 | ↓ | <0.0001 |
| SCC2022 | 1-4 | 8-10 | ↓ | <0.0001 |
|  | 5-7 | 8-10 | ↓ | <0.0001 |
| LOWSCCACHIEVABLE | ≤ 3 | 4 | ↓ | 0.0016 |
|  | ≤ 3 | 5 | ↓ | <0.0001 |
|  | 4 | 5 | ↓ | 0.0016 |
| HIGHSCCFROM | Milking process / practices | Housing | ↓ | 0.0021 |
|  | Milking process / practices | Older cows | ↑ | <0.0001 |
|  | Older cows | Sources not on current list / ‘Other’ sources | ↓ | 0.038 |
|  | Older cows | Unknown sources | ↓ | 0.032 |
| SCCADVICE | Advisory services | Veterinary professional | ↓ | 0.0013 |
|  | Advisory services | Peer to peer communication | ↓ | 0.0001 |
|  | Advisory services | Self-directed learning | ↓ | 0.042 |
|  | Advisory services | Veterinary professional and advisory services | ↓ | 0.0013 |
|  | Advisory services | Veterinary professional and peer to peer communication | ↓ | 0.0025 |
|  | Advisory services | Veterinary professionals and self-directed learning | ↓ | 0.0040 |
|  | Advisory services | Veterinary professional, advisory services, and peer to peer communication | ↓ | <0.0001 |
|  | Advisory services | Veterinary professional, advisory services, and self-directed learning | ↓ | <0.0001 |
|  | Advisory services | Veterinary professional, peer to peer communication, and self-directed learning | ↓ | 0.0058 |
|  | Advisory services | Veterinary professional, advisory services, peer to peer communication and self-directed learning | ↓ | 0.0035 |
|  | Advisory services | Veterinary professional | ↓ | 0.0013 |
|  | Peer to peer communication | Advisory services and peer to peer communication | ↑ | 0.0096 |
|  | Veterinary professional and self-directed learning | Veterinary professional, advisory services, and self-directed learning | ↓ | 0.049 |
|  | Advisory services and peer to peer communication | Veterinary professional, advisory services, and self-directed learning | ↓ | 0.0035 |
|  | Advisory services and peer to peer communication | Veterinary professional, advisory services, and peer to peer communication | ↓ | 0.0031 |
|  | Veterinary professional, advisory services, and peer to peer communication | Veterinary professional | ↑ | 0.036 |
|  | Veterinary professional, advisory services, and self-directed learning | Advisory services, peer to peer communication, and self-directed learning | ↑ | 0.0036 |
|  | Veterinary professional, advisory services, and self-directed learning | Veterinary professional | ↑ | 0.037 |
